# Dynamic control of Argonautes by a rapidly evolving immunological switch

**DOI:** 10.1016/j.cub.2025.05.039

**Published:** 2025-06-09

**Authors:** Chee Kiang Ewe, Guy Teichman, Shir Weiss, Maximilian M.L. Knott, Sarit Anava, Hila Gingold, Mario Bardan Sarmiento, Emily Troemel, Oded Rechavi

**Affiliations:** 1 School of Neurobiology, Biochemistry and Biophysics, Wise Faculty of Life Sciences & Sagol School of Neuroscience, Tel Aviv University, Tel Aviv 6997801, Israel; 2 Department of Cell and Developmental Biology, University of California, San Diego, San Diego, CA 92093, USA; 3 Lead contact

## Abstract

Small RNAs (sRNAs), coupled with Argonaute proteins (AGOs), regulate diverse biological processes, including immunity against nucleic acid parasites. *C. elegans* possesses an expanded repertoire of at least 19 AGOs functioning in an intricate gene regulatory network (GRN). However, the regulation of AGOs and how their functions adapt to genetic or environmental perturbations remains incompletely understood. Here, we report that PALS-22, a member of an unusually expanded protein family in *C. elegans*, acts as a negative regulator of antiviral RNAi involving the RIG-I homolog. Loss of *pals-22* enhances the silencing of transgenes and endogenous double-stranded RNAs (dsRNAs). We found that PALS-22 normally suppresses the expression of two AGOs, VSRA-1 and SAGO-2, which are activated by the bZIP transcription factor ZIP-1. When *pals-22* is eliminated, *vsra-1* and *sago-2* are upregulated. These AGOs, in turn, play key roles in defense against foreign genetic elements and intracellular pathogens, respectively. Surprisingly, although immune genes functioning in the intracellular pathogen response (IPR) are upregulated in *pals-22* mutants, removing SAGO-2 or the RNA-dependent RNA polymerase RRF-3 in these mutants downregulates these genes. This observation appears to contrast with the typical gene-silencing role of small interfering RNAs (siRNAs). Finally, the analysis of *C. elegans* wild isolates and lab reference strains reveals that PALS-22 regulates several germline AGOs, affecting germline mortality and transgenerational epigenetic inheritance. In summary, PALS-22 is a key genetic node that balances the trade-off between immunity and germline health by modulating the functions of different AGOs, thereby shaping the outputs of the RNAi machinery and the dynamics of epigenetic inheritance.

## In brief

Ewe et al. demonstrate that the activities of Argonaute proteins in *Caenorhabditis elegans* are regulated by an immune regulator, PALS-22. They find that PALS-22 modulates RNAi to balance the trade-off between immunity against foreign elements and “autoimmunity,” marked by aberrant silencing of self-genes and heritable germline defects.

## Graphical Abstract

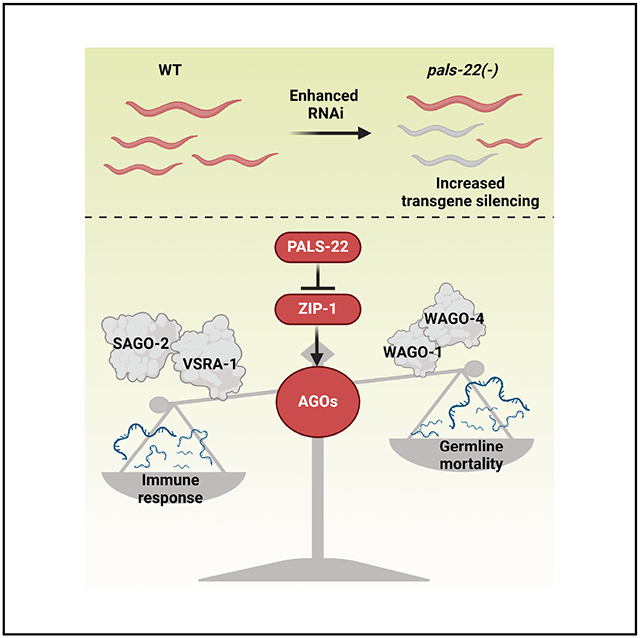

## INTRODUCTION

Non-coding small RNAs (sRNAs) play a pivotal role in gene regulation and influence most, if not all, aspects of biology. The central effectors of sRNAs are AGOs, characterized by their PAZ and PIWI domains.^1–5^ *C. elegans* contains a highly expanded RNAi network compared with many other animals, featuring at least 19 functional AGO proteins: 7 in the AGO clade, 1 in the PIWI clade, and 11 in the worm-specific Argonautes (WAGO) clade, many of which are only partially characterized.^1,2,6^ AGOs engage different classes of sRNAs, including microRNAs (miRNAs), piwi-interacting RNAs (piRNAs), and small interfering RNAs (siRNAs). Once loaded onto an AGO to form an RNA-induced silencing complex (RISC), sRNAs guide the complex to sequence-complementary RNAs, targeting them for silencing.^3^

Different AGOs exhibit distinct target repertoires, and, in many cases, it is not clear how their specificity in loading particular sRNAs is determined. Primary AGOs, including RDE-1, ERGO-1, and PRG-1, associate with exogenous siRNAs, endogenous 26G sRNAs, and 21U piRNAs, respectively.^7–10^ Upon target recognition, they trigger the production of abundant amplified 22G “secondary” sRNAs by the RNA-dependent RNA polymerase (RdRP) RRF-1.^9,11,12^ The 22G sRNAs generated by RRF-1 are loaded onto WAGOs, such as HRDE-1, PPW-1, PPW-2, and WAGO-1, that direct silencing of foreign RNA and also endogenous genes.^1,2,6^ Unusually, although most AGO-bound sRNAs silence the expression of their targets, 22G RNAs produced by RdRP EGO-1 are loaded onto CSR-1, which can instead “license” gene expression in the germ line.^13–17^ It is currently unclear how EGO-1 targets are selected, although its subcellular localization to specialized germ granule compartments may play a role.^18^

In many animals, loss of PIWI or other AGOs leads to infertility and lethality, in part due to elevated transposon mobilization and chromosomal segregation defects in the gametes and developing embryos.^19^ In *C. elegans,* CSR-1 is required for proper chromosomal pairing, segregation, and early embryonic development.^20–23^ Animals lacking *csr-1* are nearly sterile and produce few arrested embryos.^23^ On the other hand, mutants lacking the piRNA pathway or WAGOs do not show overt developmental defects but do exhibit progressive sterility in the presence of heat stress (termed mortal germline “Mrt” phenotype) likely due to transgenerational accumulation of epigenetic “baggage” and dysregulation of histone genes, rRNA, and transposable elements.^24–26^ Interestingly, a recent study demonstrated that many *C. elegans* wild isolates showed natural variation in Mrt phenotype, which can be rescued by associating the animals with their natural microbiome and pathogens, highlighting the role of environmental factors in the regulation of transgenerational epigenetic inheritance mechanisms.^27^ Although the mechanism of genomic surveillance is extensively studied in the germ line, the regulation of genomic parasites in the soma is less well understood. In the soma, the regulation of repetitive and transposable elements is far more relaxed than in the germ line. The elevated transposon mobilization in the soma was suggested to contribute to phenotypic plasticity and cellular heterogeneity, particularly in the nervous system.^28–31^ On the other hand, uncontrolled somatic transposition has been linked to various human diseases, such as psychiatric disorders,^32,33^ neurodegenerative diseases,^34,35^ and cancer.^36^

**Figure 1.**
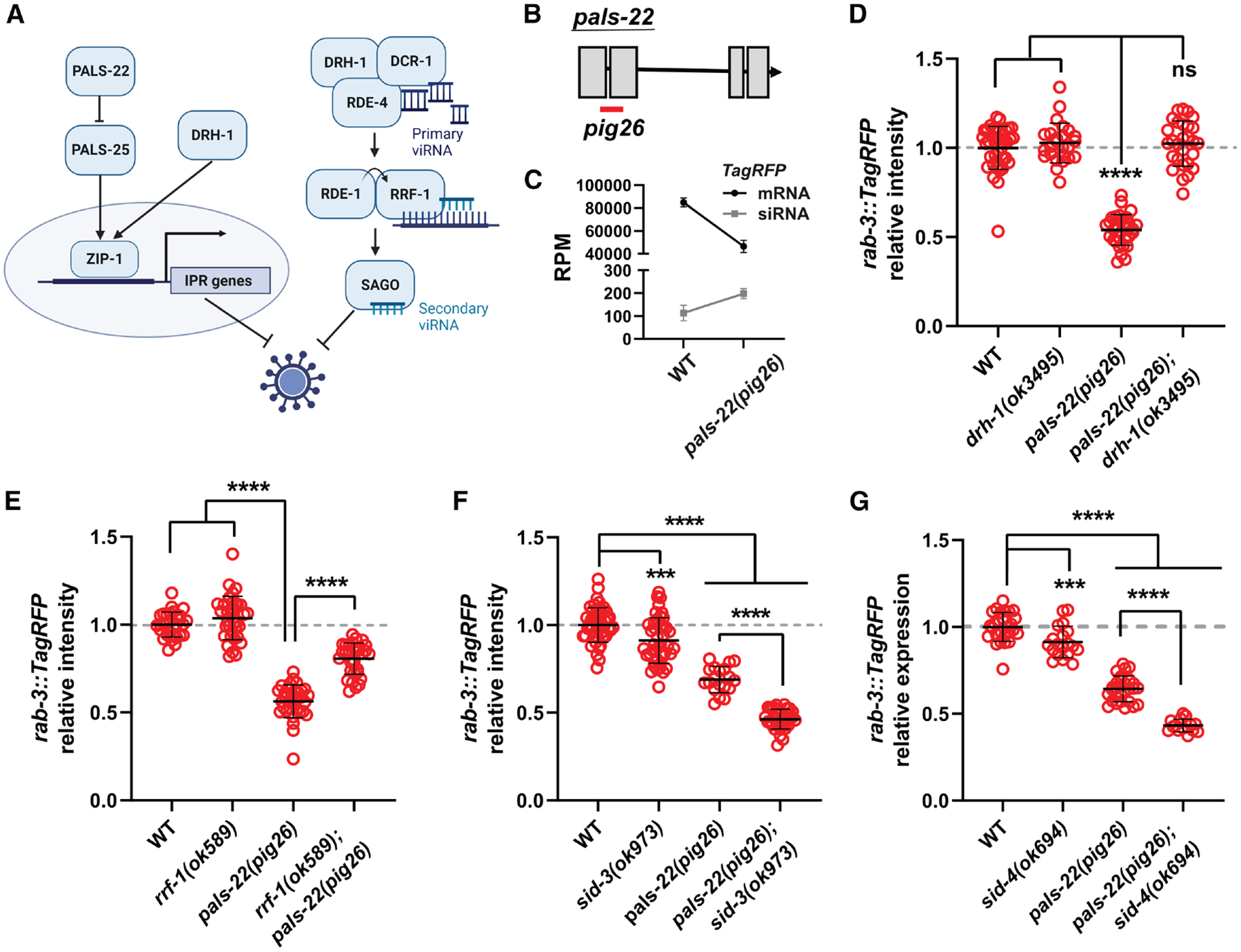
The antiviral RNAi pathway and conserved kinase signaling regulate the expression of repetitive transgenes (A) IPR and RNAi defense against viral pathogens in *C. elegans*. (B) *pig26* allele contains a 253-bp deletion in *pals-22*, indicated by the red bar. (C) mRNA and antisense siRNA reads mapped to *otIs356 [rab-3p(prom1)::2xNLS::TagRFP]* repetitive transgene in wild-type and *pals-22(pig26)* animals. RPM, reads per million. (D and E) Requirement of DRH-1 and RRF-1 for transgene silencing in *pals-22(pig26)*. (F and G) Interactions between SID-3, SID-4, and PALS-22 in transgene silencing. For (D)–(G), each data point represents an animal. *n* ≥ 14. Statistical significance was determined by one-way ANOVA followed by pairwise two-tailed unpaired t tests. ns, not significant (*q* > 0.05); ***q ≤ 0.001; ****q ≤ 0.0001. Error represents mean ± SD. See also Figure S1.

Among their many important functions, sRNA pathways play a crucial role in immunity in plants and animals.^37–39^ *C. elegans* lacks nuclear factor κB (NF-κB) signaling and canonical adaptive immunity; however, it contains homologs of RIG-I-like receptor (RLR), which are conserved RNA sensors that detect viral infection.^40^ Together with the Dicer protein DCR-1 and the double-stranded RNA (dsRNA)-binding protein RDE-4, DRH-1/RIG-I forms an antiviral complex that processes viral RNAs into 23-nt-long primary “viRNAs.” These viRNAs are then loaded onto the primary AGO RDE-1, which recruits RRF-1 to the viral target transcripts to generate secondary siRNAs, which are subsequently loaded onto WAGOs, such as SAGO-2, to promote degradation of complementary viRNA targets (Figure 1A).^1,41–44^

Aside from using RNAi to target viruses, *C. elegans* also mounts a transcriptional response against intracellular pathogens such as Orsay virus (the sole known natural virus of *C. elegans*). This response has similarities with transcriptional induction caused by the microsporidian *Nematocida parisii*—an intracellular fungal pathogen.^42,43,45–50^ This intracellular pathogen response (IPR) involves an unusually expanded *pals* (protein-containing ALS2CR12 signature) gene family. *C. elegans pals* genes fall into two classes: (1) IPR genes induced by infection, such as *pals-5* and *pals-14*, and (2) regulators of IPR genes, like *pals-22* and *pals-25* (see below). It is noteworthy that *C. elegans* contains at least 39 copies of *pals* genes, compared with 8 in its cousin *C. briggsae*.^51^ This rapid proliferation of protein-coding genes is extraordinary and may reflect the intense host-pathogen arms race that drives escalated diversification of innate immunity as well as contribute to extensive natural variation in viral susceptibility among closely related nematode species.^42,52,53^

One of the regulator *pals* genes, *pals-22*, is known to function as a repressor of the IPR by inhibiting the activator *pals-25*. Loss of *pals-22* in *C. elegans* enhances their resistance to viral and microsporidian pathogens in a *pals-25*-dependent manner.^47^ Interestingly, DRH-1 may also activate IPR gene expression independently of RNAi factors, much like mammalian RLR.^54,55^ Upon viral infection, DRH-1 induces upregulation and nuclear localization of the ZIP-1/bZIP transcription factor, triggering IPR gene expression and antiviral defense (Figure 1A), similar to loss of *pals-22*.^40,43,56^ Curiously, eliminating *pals-22* causes enhanced silencing of repetitive transgenes and enhanced exogenous RNAi response (classical “Eri” phenotypes),^47,51,57^ poising PALS-22 at a critical node that regulates a broad range of genomic parasites, although the mechanism of action of PALS-22 in the RNAi pathways remains elusive.

## RESULTS

### Loss of *pals-22* enhances repetitive transgene silencing via the antiviral RNAi pathway

To study the roles of PALS-22 in the RNAi pathways, we used CRISPR-Cas9 to generate a deletion allele of *pals-22* (designated *pig26*) (Figure 1B; see STAR Methods). We found that *pals-22(pig26)* exhibits many phenotypes of previously characterized loss-of-function mutations of *pals-22*, including increased repetitive transgene silencing (Figures 1C, S1A, and S1B),^51^ enhanced resistance to Orsay virus and *N. parisii* infection (see below),^47^ decreased brood size (Figure S1C), and age-dependent locomotory defects (Figure S1D).^51^

LIN-35/Rb and the DREAM/Muv B complex repress germ cell fate in the soma. Inactivating *lin-35* leads to a partial soma-to-germline transformation, as evidenced by the presence of ectopic germline P granules and enhanced RNAi.^58^ Intriguingly, a recent study demonstrated that the loss of *lin-35/Rb* induced a transcriptional response similar to that of *pals-22(−)* mutants and *N. parisii*-infected worms.^59^ Therefore, we asked whether the enhanced transgene-silencing activity in *pals-22(pig26)* is the result of de-repression of germline RNAi in somatic cells, as observed in *lin-35(−)* mutants. However, we did not observe obvious misexpression of the germline P granule component PGL-1 in the soma of *pals-22(pig26)* mutants (Figure S1E).

The nuclear RNAi factor NRDE-3 directs deposition of H3K9 methylation and induces transcriptional silencing of repetitive foreign elements.^60^ However, we found that enhanced transgene silencing in *pals-22(pig26)* does not depend on NRDE-3. Although NRDE-3 localizes to the cytoplasm in many Eri mutants due to absence of endo-siRNAs,^61^ it remains prominently nuclear in *pals-22(pig26)* mutants (Figure S1F). Additionally, knocking out *nrde-3* or *set-25/HMT* does not affect *pals-22 (pig26)* transgene-silencing phenotype (Figure S1G). On the other hand, knocking out *drh-1* or *rrf-1* strongly suppresses the silencing phenotype of *pals-22(pig26)* mutants (Figures 1D and 1E), suggesting that the loss of *pals-22* triggers post-transcriptional cytoplasmic silencing of repetitive element by the antiviral RNAi pathway. Concordant with RNAi-mediated silencing of transgenes, we observed a significant upregulation of siRNAs targeting *TagRFP* (*otIs356* integrated repetitive transgene)^62^ in *pals-22(pig26)* mutants (log_2_FC = 0.70, DESeq2 *q* = 0.02; Figure 1C). In line with the hyperactive RNAi phenotype following the loss of *pals-22*, genes associated with the Gene Ontology (GO) term “regulatory ncRNA-mediated post-transcriptional gene silencing” are significantly enriched among those with increased expression in *pals-22(pig26)* mutants versus wild-type worms (GO: 0035194; 22 observed and 8.36 expected; Fisher’s exact test, *q* = 0.005) (Figure S1H). Indeed, *drh-1*, along with genes encoding core components of the antiviral RNAi pathway, such as *rde-1*, *rde-4*, and *rrf-1*, are upregulated in *pals-22(pig26)* mutants (Figures S1I and S1J). Additional genes encoding factors involved in the amplification of secondary siRNAs, including *ekl-1*, *drh-3*, *rsd-2*, and *rde-12*, also showed elevated expression in *pals-22* mutants relative to the wild-type worms (Figures S1I and S1J). On the other hand, genes encoding RNAi factors specifically act in the 26G endo-siRNA pathway are not differentially expressed in *pals-22(pig26)*, except for *eri-6/7*, which are modestly downregulated in this mutant compared with wild type (Figure S1K).

Prior studies reported that *sid-3/TNK2*, which encodes a cytoplasmic tyrosine kinase implicated in RNA import and endosomal trafficking, is critical for Orsay virus infection.^46,63,64^ Eliminating SID-3 enhances resistance to viral infection in worms, likely due to a defect in viral entry.^46^ Interestingly, we found that knocking out *sid-3* leads to a modest but statistically significant drop in transgene expression (*q* = 0.0004; Figure 1F). Moreover, *pals-22(pig26)*; *sid-3(ok973)* double mutants show stronger silencing than either of the single mutants (Figure 1F). In humans, TNK2 is known to interact with an adaptor protein, NCK1, to promote virus entry and facilitate subsequent proper endocytic trafficking of the viral particles.^46,65^ Consistently, knocking out *sid-4/NCK1* (also named *nck-1*) enhances *pals-22(−)*’s transgene-silencing phenotype (Figure 1G). Together, our results demonstrate that antiviral immunity and repetitive DNA are co-regulated by PALS-22 and a conserved kinase signaling pathway. Further investigation is warranted to elucidate the mechanism by which SID-3 and SID-4 promote the expression of repetitive foreign genetic elements.

### PALS-22 represses expression of the Argonaute protein VSRA-1

**Figure 2.**
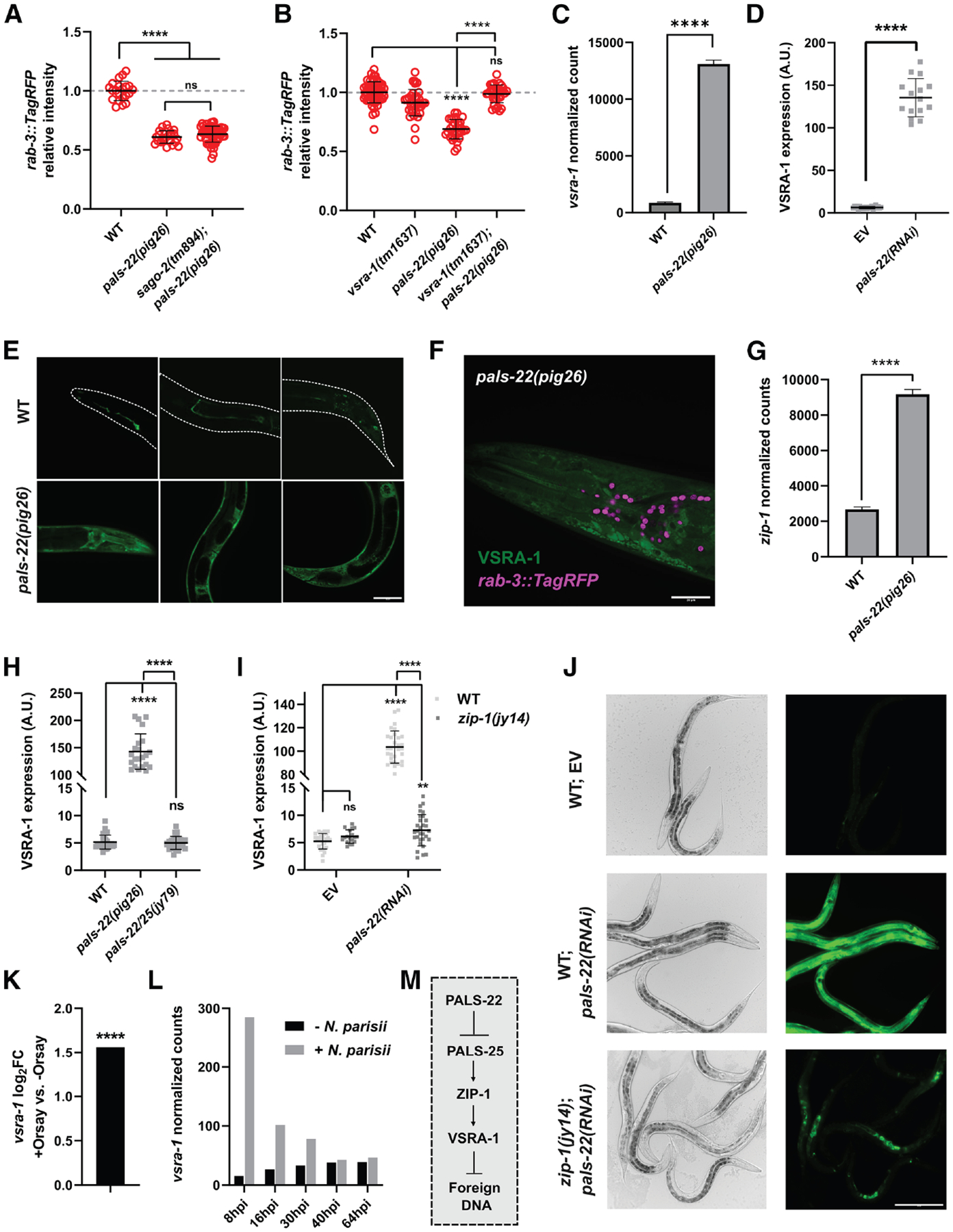
PALS-22 represses VSRA-1 (A) Requirement of SAGO-2 for transgene silencing in *pals-22(pig26)*. (B) Requirement of VSRA-1 for transgene silencing in *pals-22(pig26)*. Each data point represents an animal. *n* ≥ 21. Statistical significance was determined by one-way ANOVA followed by pairwise two-tailed unpaired t tests. (C) Expression of *vsra-1* in wild-type and *pals-22 (pig26)*. Relative log expression (RLE) normalized mRNA read counts are shown. (D) Expression of endogenous VSRA-1 reporter in wild-type and *pals-22(RNAi)* animals. Day 1 adults were scored. *n* ≥ 15. a.u., arbitrary unit. Statistical significance was determined by Mann-Whitney test. (E) Tissue localization of VSRA-1 in wild-type and *pals-22(pig26)* day 1 adults. Scale bar, 50 μm. (F) Accumulation of VSRA-1 in the cytoplasm in *pals-22(pig26)*. Neuronal nuclei are marked by *otIs356 [rab-3p::TagRFP]*. Scale bar, 20 μm. (G) Expression of *zip-1* in wild-type and *pals-22 (pig26)*. RLE normalized mRNA read counts are shown. (H) Expression of VSRA-1 endogenous reporter in *pals-22(pig26)* compared with *pals-22/25(jy79)* animals. (I and J) Expression of VSRA-1 endogenous reporter in *pals-22(RNAi)* compared with *zip-1(jy14); pals-22(RNAi)* animals. Scale bar, 200 μm. For (H) and (I), each data point represents an animal. *n* ≥ 18. Statistical significance was determined by Kruskal-Wallis test followed by pairwise Mann-Whitney tests. (K and L) Expression of *vsra-1* during infection with Orsay virus^66^ or *N. parisii*.^45^ hpi, hours post inoculation. FPKM mRNA read counts are shown in (L). (M) A model indicating the regulation and function of VSRA-1. For all relevant panels, error represents mean ± SD. ns, not significant (*q* > 0.05); ***q ≤ 0.001; ****q ≤ 0.0001. See also Figure S2 and Table S1.

The foregoing results suggest that the antiviral RNAi response promotes transgene silencing in the soma. This observation led us to test the functions of the SAGO-2/WAGO, which has previously been shown to be important for fighting viral infections.^1,41^ However, we found that knocking out *sago-2* does not alter the expression of repetitive transgenes in both wild-type and *pals-22(pig26)* mutants (Figure 2A), suggesting that the antiviral RNAi pathway engages a different WAGO to distinguish exogenous/virus-derived RNA from repetitive transgenes.

By examining co-immunoprecipitation (coIP) mass spectrometry data of PALS-22,^67^ we noted that PALS-22 likely physically interacts with VSRA-1, a close paralog of CSR-1.^1,6^ We found that deleting *vsra-1* strongly rescues transgene silencing in *pals-22(pig26)* (Figures 2B and S2A). We found that *vsra-1* transcripts are highly upregulated in *pals-22(pig26)* (log_2_FC = 3.9; Figure 2C). Consistently, by examining an endogenous fluorescent translational reporter of VSRA-1, we found that VSRA-1 is dramatically upregulated in *pals-22(pig26)* and *pals-22(RNAi)* animals (Figures 2D–2F and S2B). This upregulation is observed across all developmental stages (Figures S2B and S2C). Moreover, although VSRA-1 is only detectable in the somatic gonad and a small number of neurons in wild-type animals (Figure 2E; Table S1), we observed that it is expressed ubiquitously in *pals-22(pig26)* mutants (Figure 2E). VSRA-1 appears to exclusively localize to the cytoplasm (Figure 2F), suggesting that VSRA-1 functions in post-transcriptional gene regulation. We hypothesized that ectopic overexpression of *vsra-1* in the *pals-22(−)* background induces silencing of repetitive transgenes. Consistent with this model, driving *vsra-1* expression under the control of a strong, ubiquitous promoter significantly downregulates transgene expression (Figure S2D).

ZIP-1/bZIP is a transcription factor that is required to induce one-third of IPR genes, acting downstream of PALS-25. Eliminating *zip-1* leads to increased susceptibility to intracellular pathogens (see Figure 1A).^56,57^ We found that *zip-1* is significantly upregulated in *pals-22(pig26)* mutants in which the IPR is constitutively activated (Figure 2G), in agreement with previous studies.^56,57^ Hence, we hypothesized that *vsra-1* may be activated by ZIP-1 downstream of PALS-25. Indeed, deleting *pals-25* suppresses *vsra-1* expression in *pals-22(pig26)* mutants (Figure 2H). Furthermore, knocking down *zip-1* by RNAi significantly downregulates *vsra-1* in *pals-22(pig26)* mutant background (Figures S2E and S2F). We observed a similar effect when *pals-22* is eliminated by RNAi in *zip-1(jy14)* mutants (Figure 2I). However, intriguingly, *vsra-1* remains upregulated in the somatic gonad of *zip-1(jy14); pals-22(RNAi)* animals (Figure 2J), suggesting that PALS-22 engages tissue-specific gene regulatory modules. The biological significance of elevated *vsra-1* expression in the somatic gonad is currently unclear, but it is worth noting that it is the only tissue, besides the intestine, that is susceptible to Orsay virus infection.^50^

Interestingly, by examining published transcriptomic data, we found that *vsra-1* is upregulated in worms infected with Orsay virus (Figure 2K)^66^ or *N. parisii* (Figure 2L).^45^ During *N. parisii* infection, this upregulation is especially pronounced at early time points that correspond to the entry and replication of the parasite inside host intestinal cells (Figure 2L).^45,49^ On the contrary, *vsra-1* expression remains unchanged in animals exposed to extracellular bacterial and other fungal pathogens.^68^ Taken together, these results indicate that VSRA-1 responds to the presence of intracellular pathogens, downstream of the PALS-25 → ZIP-1 regulatory axis and mounts an RNAi defense to mitigate the infection (Figure 2M). Supporting this notion, knocking down *vsra-1* by RNAi causes increased susceptibility to viral infection.^69^

### PALS-22 promotes the expression of endogenous dsRNAs and silences newly evolved genes

**Figure 3.**
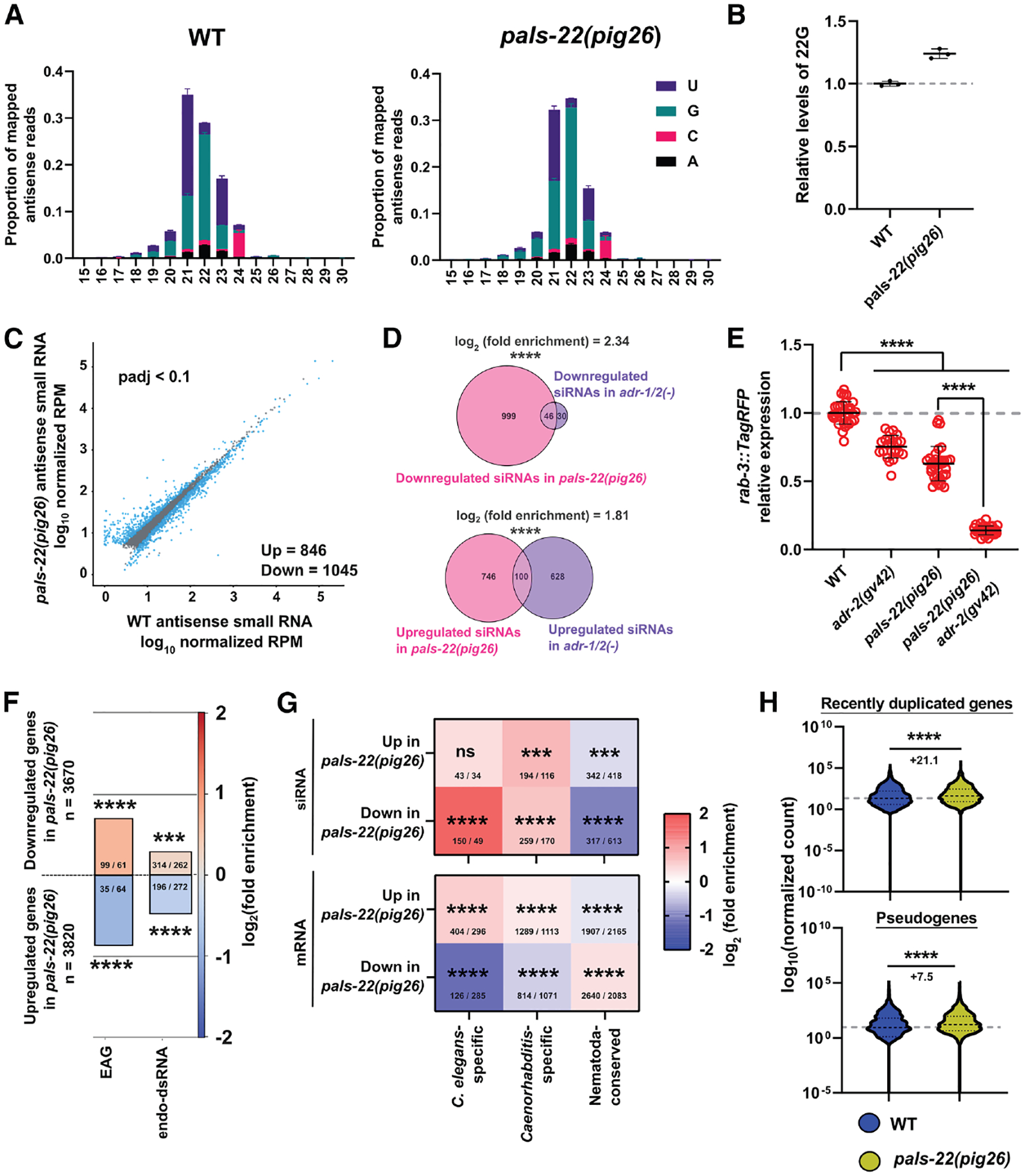
Loss of *pals-22* leads to remodeling of the sRNA pool (A) Antisense sRNA size and 5′ nt distribution in wild-type and *pals-22(pig26)*. The abundant 21Us are derived from four miRNAs, *mir-1*, *mir-71*, *mir-45*, and *mir-234*. (B) Levels of 22G siRNA in *pals-22(pig26)* normalized to wild type. (C) Differentially expressed siRNAs (DESeq2 *q* < 0.1) in *pals-22(pig26)* compared with wild type. RPM, read per million. (D) Overlaps of differentially expressed siRNAs (DESeq2 *q* < 0.1) in *pals-22(pig26)* and *adr-1/2(−)*. (E) Interactions between PALS-22 and ADR-2 in transgene silencing. Each data point represents an animal. *n* ≥ 23. Statistical significance was determined by Kruskal-Wallis test followed by pairwise Wilcoxon rank-sum tests. Error represents mean ± SD. (F) Enrichment analysis for A-to-I editing-associated genes (EAGs) and endo-dsRNA (ΔG/nt < − 0.5 kcal/mol × nt) in *pals-22(pig26)* differentially expressed mRNAs. (G) Enrichment analysis for *C. elegans*-specific, *Caenorhabditis*-specific, and Nematoda-conserved genes in *pals-22(pig26)* differentially expressed siRNAs and mRNAs. The observed/expected number of genes in each category is indicated. ns, not significant (*q* > 0.05); ***q ≤ 0.001; ****q ≤ 0.0001. (H) Expression of recently duplicated genes (*n* = 4,310) and pseudogenes (*n* = 1,354) in wild type and *pals-22(pig26)*. RLE mRNA read counts are shown. The numbers indicate differences between median normalized counts. The average log_2_FC (*pals-22(pig26)* versus wild type) of recently duplicated genes and pseudogenes are 1.03 and 1.23, respectively. Statistical significance was determined by Mann-Whitney test. ****p ≤ 0.0001. See also Figure S3, Table S2, and Data S1.

To further probe the roles of PALS-22 in somatic sRNA pathways, we sequenced sRNAs and mRNAs isolated from wild-type and *pals-22(pig26)* L1 animals. The sRNAs were cloned using a 5^′^-phosphate-independent protocol to capture both primary and secondary sRNAs (see STAR Methods). We chose to sequence L1 animals because they contain 550 somatic cells and only two primordial germ cells, and thus any transcriptional changes are likely to be indicative of somatic responses. Consistent with our previous findings that the antiviral RNAi pathway is enhanced in the absence of *pals-*22, we found that the overall levels of 22G secondary siRNAs are significantly elevated in *pals-22(pig26)* (Figures 3A and 3B).

Loss of *pals-22* leads to differential expression of many siRNAs targeting protein-coding and non-protein-coding genes (DESeq2 *q* < 0.1; Figure 3C). By taking a closer look at the gene biotypes, we found that siRNAs targeting protein-coding genes (average FC = 1.15) and lincRNAs (average FC = 1.61) are upregulated in *pals-22(pig26)*, whereas the levels of miRNAs and piRNAs remain largely unchanged (Figure S3). To gain further insights into the roles of PALS-22, we performed WormCat GO analysis^70^ on siRNAs differentially expressed in *pals-22(pig26)* mutants. Our analysis revealed an enrichment of pseudogenes in the downregulated set. Interestingly, siRNAs targeting stress response genes, including many *pals* family members, tend to be upregulated in *pals-22(pig26)* mutants (Table S2) (more below).

In *C. elegans*, zebrafish, and mammals, adenosine deaminase acting on RNA (ADAR) enzymes are required to prevent ectopic activation of the antiviral response, involving RLR, targeting self dsRNAs.^71–73^ In vertebrates, recognition of dsRNAs by MDA-5 and RIG-I triggers the interferon (IFN)-response.^72,73^ In *C. elegans*, RNA editing disrupts dsRNA formation and thus antagonizes the production of dsRNA-derived siRNAs by DCR-1.^72,74,75^ Loss of ADARs leads to transgene silencing and reduced lifespan, phenotypes also observed in *pals-22(−)* mutants (see Figure S1).^51,76^ Hence, we asked whether PALS-22 may interact with ADARs to regulate the expression of transgene-derived and endogenous dsRNAs. Supporting our hypothesis, we found that the sRNA profile of *pals-22(pig26)* significantly overlaps with that of *adr-1/2(−)* mutants (Figure 3D). Indeed, we found that PALS-22 and ADR-2 function in parallel to promote repetitive transgene expression, as the *pals-22 (pig26) adr-2(gv42)* double mutant shows a stronger silencing phenotype than either of the single mutants (Figure 3E). Moreover, many A-to-I editing-associated genes (EAGs), previously defined by Reich et al.,^74^ and endo-dsRNA containing structured introns (ΔG/nt < − 0.5 kcal/mol × nt)^77^ tend to be downregulated in *pals-22(pig26)* (Figure 3F). These findings suggest that PALS-22 prevents aberrant silencing of endogenous dsRNAs, likely by suppressing the antiviral RNAi pathway.

Evolutionarily young genes tend to be the targets of endogenous siRNA pathways.^78–80^ On the other hand, dsRNA-associated genes targeted by ADAR are enriched among conserved, essential genes.^77 ,81^ We found that the loss of *pals-22* causes downregulation of siRNAs targeting newly evolved genes, such as *C. elegans*-specific genes and genes that have no orthologs outside of the *Caenorhabditis* genus (Figure 3G; see also Table S2). Concordantly, we found a significant enrichment for newly evolved genes, including pseudogenes and young duplicated genes, among the mRNAs that are upregulated in *pals-22(pig26)* (Figures 3G and 3H). In contrast, downregulated mRNAs in *pals-22(pig26)* are enriched for older genes that are conserved across the Nematoda phylum (Figure 3G). Hence, PALS-22 appears to block the expression of certain young genes while promoting the expression of a subset of conserved genes.

Collectively, our findings indicate that the loss of *pals-22* affects the sRNA landscape, including upregulation of 22G siRNAs, and imparts a variety of gene regulatory outcomes on targets’ transcripts with distinct features—possibly including secondary structures, number of introns, and divergent splicing signals—recognized by the RNAi machinery.^78,80^

### Dual effects of RRF-3-dependent endo-siRNAs on IPR gene expression

**Figure 4.**
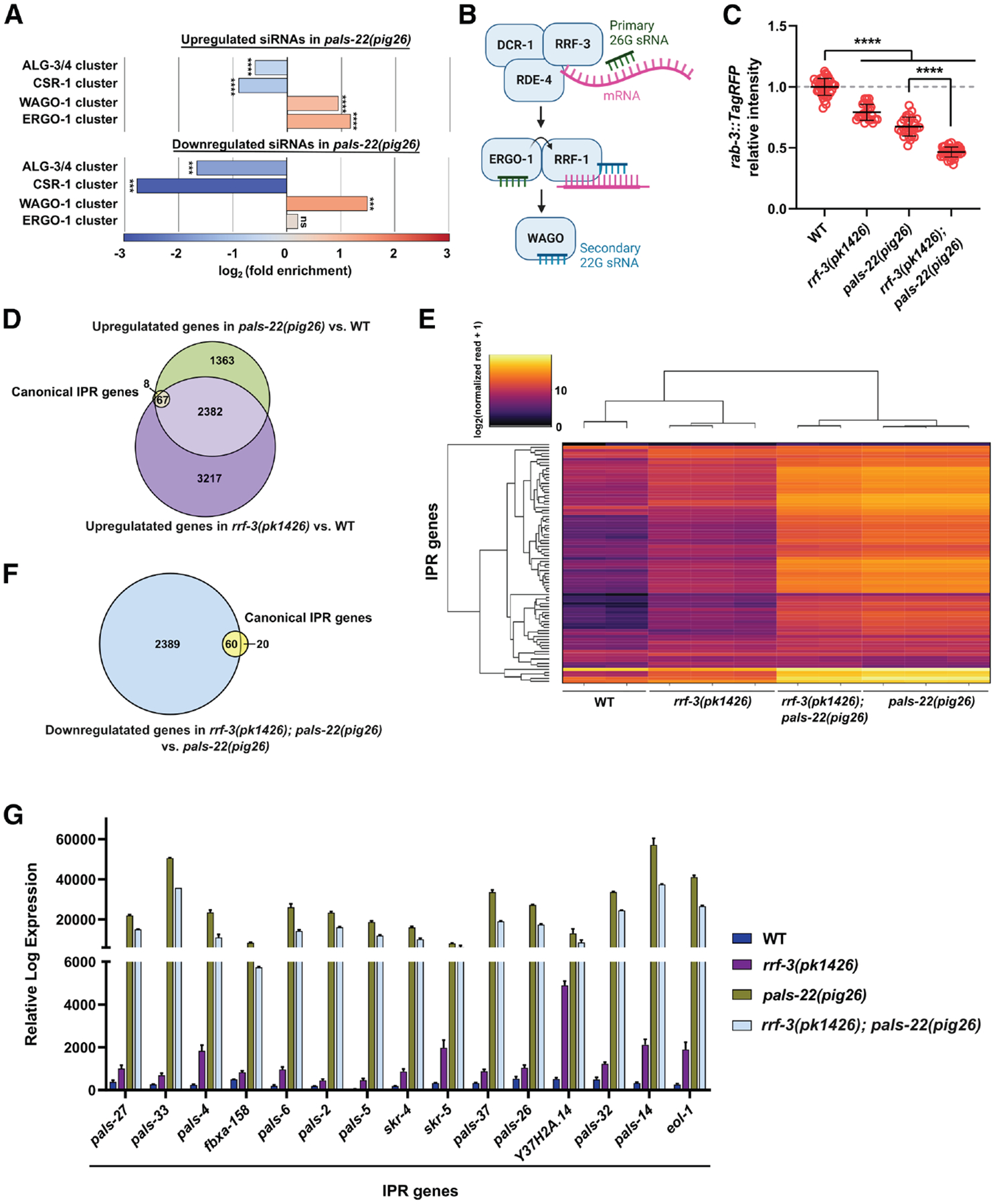
Dual roles of endo-siRNA in regulating IPR gene expression (A) Enrichment analysis for AGO-associated sRNAs in *pals-22(pig26)* differentially expressed siRNAs. Endo-siRNA-associated AGOs are divided into four clusters.^1^ (B) Overview of the 26G endo-siRNA pathway. (C) Interactions between PALS-22 and RRF-3 in transgene silencing. *n* ≥ 25. Statistical significance was determined by Kruskal-Wallis test followed by pairwise Wilcoxon rank-sum tests. ****q ≤ 0.0001. (D) Venn diagram showing the overlap between 80 canonical IPR genes and genes upregulated in *pals-22(pig26)* and *rrf-3(pk1426)* mutants compared with wild type. DESeq2 *q* < 0.1. (E) Hierarchical clustergram plot visualizing the expression of IPR genes in wild type, *rrf-3 (pk1426)*, *rrf-3(pk1426); pals-22(pig26)*, and *pals-22(pig26)* using Euclidean distance metric and average linkage. (F) Venn diagram showing the overlap between 80 canonical IPR genes and genes downregulated in *rrf-3(pk1426); pals-22(pig26)* double mutants compared with *pals-22(pig26)* single mutants. DESeq2 *q* < 0.1. (G) Examples of IPR genes upregulated in *rrf-3 (pk1426)* compared with wild type but downregulated in *rrf-3(pk1426); pals-22(pig26)* compared with *pals-22(pig26)*. RLE normalized mRNA read counts are shown. For (C) and (G), error represents mean ± SD. See also Figure S4 and Data S2.

In *C. elegans*, genes generating siRNAs can be classified based on the targeting AGOs (Figure 4A).^1^ Some of these siRNAs induce the production of secondary siRNAs, which then bind to other AGOs, although we did not differentiate these specific categories. We noted that differentially expressed siRNAs in *pals-22(pig26)* tend to associate with the “WAGO cluster” composed of WAGO-1, PPW-2, HRDE-1, and PPW-1, which are major regulators of repetitive and transposable elements.^1^ Furthermore, upregulated siRNAs in *pals-22(pig26)* are enriched for the “ERGO-1 cluster” (composed of SAGO-2, SAGO-1, ERGO-1, and NRDE-3) targets, which are largely associated with immune, defense, and stress responses (Figure 4A).^1^

RRF-3 is an RdRP that functions in the endo-siRNA pathway, upstream of ERGO-1, to generate primary 26G sRNAs using the target mRNA as template (Figure 4B).^2^ Consistent with previous findings,^82^ knocking out *rrf-3* promotes silencing of repetitive transgenes. We found that loss of *pals-22* further enhances the *rrf-3* transgene-silencing phenotype (Figure 4C), indicating that RRF-3 and PALS-22 function in parallel to antagonize RNAi against foreign genes.

Next, to probe how PALS-22 and the endo-siRNA pathway regulate immunity, we performed RNA sequencing (RNA-seq) analysis and examined IPR gene expression in *rrf-3(−)* and *pals-22(−)* mutants. As expected, eliminating *pals-22* causes a dramatic increase in IPR gene expression compared with wild type.^47,83^ Similarly, IPR genes are upregulated in *rrf-3(pk1426)* compared with wild type (DESeq2 *q* < 0.1), albeit to a lesser extent, suggesting that the 26G endo-siRNA pathway normally silences IPR genes (Figures 4D and 4E).

As IPR genes are strongly upregulated in *pals-22(pig26)*, we expected to observe a decrease in the levels of sRNAs that target these genes. To our surprise, we witnessed that exact opposite: sRNAs targeting IPR genes are significantly upregulated in *pals-22(pig26)* mutants (DESeq2 *q* < 0.1; Figure S4A) and are positively correlated with the expression of IPR transcripts (Spearman’s ρ = 0.34, slope = 0.47) (Figure S4B), in contradiction to the typical gene-silencing role of siRNAs. We sought to further investigate this unusual pattern of gene regulation by examining the interactions between PALS-22, RRF-3, and downstream WAGOs in this process (see below).

Intriguingly, although IPR genes are upregulated in each of the single mutants, 60 out of 80 IPR genes are significantly downregulated in the *rrf-3(pk1436); pals-22(pig26)* double mutant compared with *pals-22(pig26)* (DESeq2 *q* < 0.1; Figures 4E–4G). Similarly, knocking out *ergo-1* in *pals-22(RNAi)* animals causes downregulation of IPR genes (Figure S4C). However, we found that eliminating *rrf-3* in either wild-type or *pals-22 (pig26)* animals does not overtly affect their resistance to Orsay virus or *N. parisii* (Figures S4D–S4F).

Together, our results suggest that the sRNA pathway may exert opposing effects on IPR gene expression: in the wild-type background, the 26G endo-siRNA pathway, acting through RRF-3, represses IPR gene expression; however, in the absence of *pals-22*, ERGO-1 and RRF-3 appear to upregulate IPR genes. It is important to note that we cannot rule out the possibility that RRF-3 engages in additional gene regulatory interactions that indirectly influence IPR gene expression.

### PALS-22 regulates a negative and positive gene expression switch via SAGO-2

**Figure 5.**
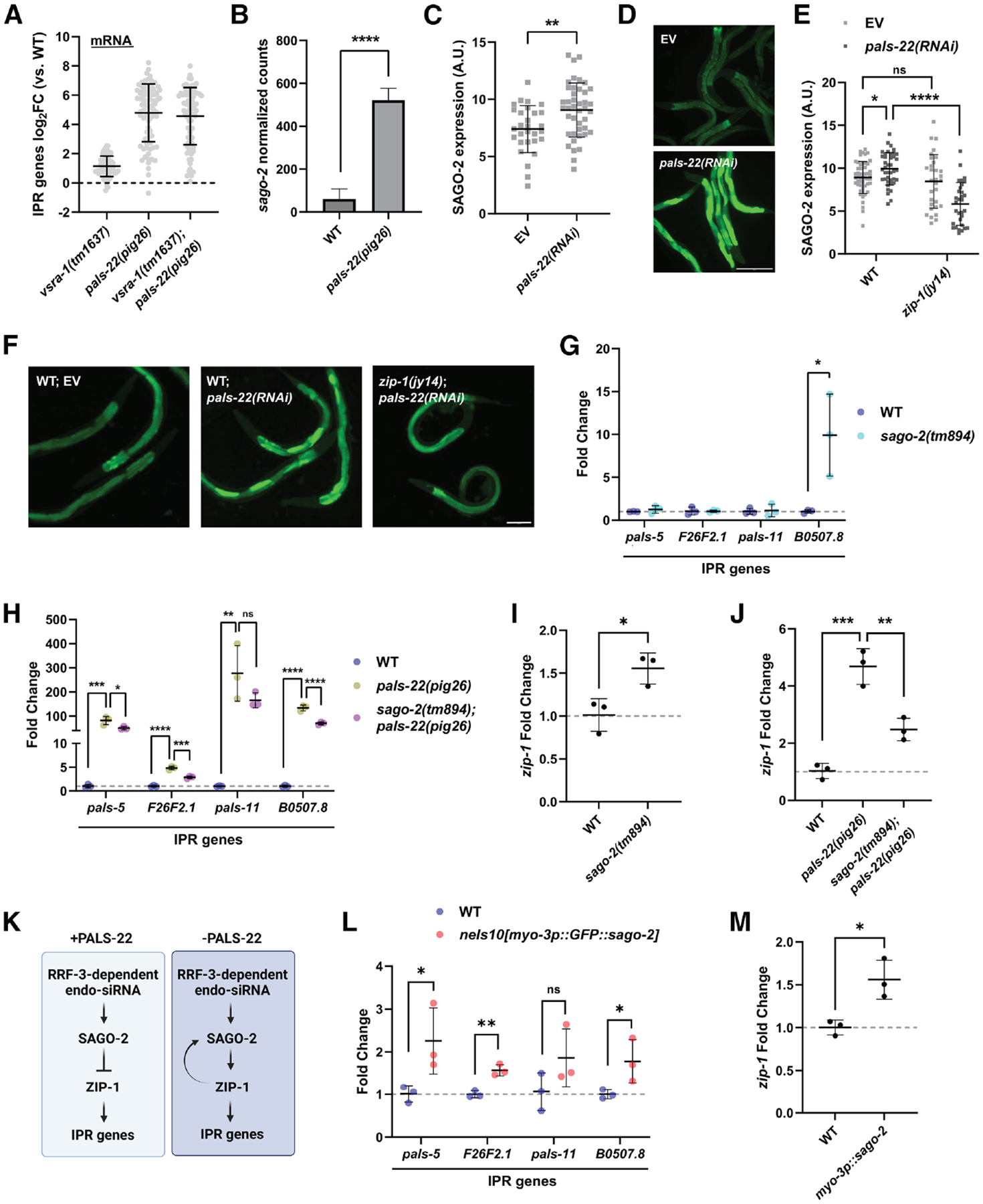
SAGO-2 regulates IPR gene expression (A) Fold change in expression of 80 canonical IPR genes in *vsra-1(tm1637)*, *pals-22(pig26)*, and *vsra-1(tm1637); pals-22(pig26)*, relative to wild type. (B) The expression of *sago-2* in *pals-22(pig26)*. RLE normalized mRNA read counts are shown. (C and D) The expression of SAGO-2 endogenous reporter in animals treated with empty vector (EV) and *pals-22* RNAi. *n* ≥ 29. Scale bar, 200 μm. (E and F) The expression SAGO-2 endogenous reporter in wild-type and *zip-1(jy14)* animals treated with EV or *pals-22* RNAi. *n* ≥ 31. Scale bar, 100 μm. (G) The expression of IPR genes in wild type and *sago-2(tm894)*, as measured by qPCR. Statistical significance was determined by Mann-Whitney test. (H) The expression of IPR genes in wild type, *pals-22(pig26)*, *sago-2(tm894); pals-22(pig26)*, as measured by qPCR. All strains contain *otIs356 [rab-3p::TagRFP]* transgene. Statistical significance was determined by Kruskal-Wallis test followed by pairwise Wilcoxon rank-sum tests. (I) The expression of *zip-1* in wild type and *sago-2 (tm894)*. (J) The expression of *zip-1* in wild type, *pals-22 (pig26)*, *sago-2(tm894); pals-22(pig26)*, as measured by qPCR. All strains contain *otIs356 [rab-3p::TagRFP]* transgene. (K) A model indicating the role of endo-siRNA in either inhibiting or promoting *zip-1* and IPR gene expression, depending on the activity of PALS-22. (L and M) The expression of IPR genes and *zip-1* in wild type and animals carrying *neIs10 [myo-3:: GFP::sago-2]*. For (C), (I), (L), and (M), statistical significance was determined by two-tailed unpaired t test. For (E) and (J), statistical significance was determined by one-way ANOVA followed by pairwise two-tailed unpaired t tests. For all relevant panels, ns, not significant (*q* > 0.05); *q ≤ 0.05; **q ≤ 0.01; ***q ≤ 0.001; ****q ≤ 0.0001. Error represents mean ± SD. See also Figures S5–S7.

As we have shown that VSRA-1 silences transgenes in *pals-22(−)* mutants, we next asked whether VSRA-1 may function downstream of PALS-22 and RRF-3 to regulate IPR gene expression. We found that IPR genes are modestly upregulated in *vsra-1 (tm1637)* single mutants; however, unlike *rrf-3*, knocking out *vsra-1* does not alter IPR gene expression in *pals-22(pig26)* mutants (Figure 5A).

In search of a different AGO that could regulate IPR gene expression, we noted that *sago-2*, like *vsra-1*, is significantly upregulated when *pals-22* is eliminated by a null mutation or RNAi (Figures 5B–5D). Additionally, we observed that some sRNAs associated with SAGO-2 are upregulated in *pals-22(pig26)* mutants (Figures S5A and S5B). Similar to *vsra-1*, we found that PALS-22 normally represses *sago-2* expression by suppressing the PALS-25 → ZIP-1 genetic module. Although loss of *zip-1* alone does not affect *sago-2* expression (Figures 5E and S5C), knocking out *zip-1* in *pals-22(RNAi)* animals downregulates *sago-2* (Figures 5E and 5F).

To search for additional regulators of *sago-2*, we leveraged the extensive natural variation of *sago-2* expression among *C. elegans* wild isolates (Figure S6A) and performed an eQTL analysis on the CaeNDR platform.^84–87^ This analysis revealed a local QTL on chromosome (chr)I and two distant QTLs on chrII and chrIII (Figures S6B and S6C). We subsequently performed a targeted RNAi screen, focusing on candidates in chrII, and found that knocking out *cpna-1/F31D5.3* by RNAi in the wild-type background modestly reduces *sago-2* expression (Figure S6D). Interestingly, *cpna-1* encodes a copine domain protein, which has been implicated in immune responses from plants to mammals.^88–91^ CPNA-1 has previously been shown to physically interact with DCR-1,^92^ suggesting the potential regulation of *sago-2* by the RNAi machinery. However, a study has found that null mutations in *cpna-1* cause severe embryonic lethality and elongation defects,^93^ complicating efforts to study its pleiotropic roles in RNAi and immunity.

Although we found that SAGO-2 plays no role in transgene silencing in *pals-22(−)* mutants, it appears to target a subset of IPR genes (Figures 5G and S5D). Notably, as observed in *rrf-3* mutants, knocking out *sago-2* alone promotes IPR gene expression; however, eliminating *sago-2* in the *pals-22(pig26)* background leads to a downregulation of IPR gene expression (Figures 5G and 5H). This may relate to a previous observation that mutation of *rde-4*, a gene encoding a dsRNA-binding protein acting in the Dicer complex, modestly reduces IPR gene expression in a *pals-22(−)*.^83^ SAGO-2 may directly bind to and regulate IPR genes and/or influence *zip-1* expression, which, in turn, activates IPR gene expression. Indeed, *zip-1* appears to be a target of SAGO-2 (IP enrichment = 2.27),^1^ and its expression is differentially regulated by SAGO-2 and PALS-22: loss of *sago-2* alone leads to upregulation of *zip-1*, whereas its removal in the *pals-22(pig26)* background results in downregulation—a pattern we repeatedly observed in IPR genes (Figures 5I and 5J). These results demonstrate that SAGO-2 normally represses IPR gene expression; however, in the absence of *pals-22*, SAGO-2 may positively regulate IPR genes, at least partly by upregulating *zip-1* (Figures 5K and S7).

Given that *sago-2* expression is elevated in the *pals-22(−)* mutant, we hypothesize that SAGO-2 may either negatively or positively regulate the IPR, depending on its expression levels. Consistent with this notion, SAGO-2 overexpression in the muscles upregulates IPR genes and *zip-1* (Figures 5L and 5M). Similar results were observed when we overexpressed *sago-2* under the control of its native promotor (Figures S5E and S5F), suggesting that its regulatory function is influenced by molecular stoichiometry.

### Rapid evolution of the PALS-22/PALS-25 locus may underlie natural variation in the Mrt phenotype

Previous studies have shown that PALS-22 inhibits the IPR transcriptional program, which includes a suite of *pals* genes as well as the cullin-RING ubiquitin ligase components, promoting proteostasis capacities and resistance to infection with intracellular pathogens, such as *N. parisii*.^40 ,45,67,83,94,95^ Building on these findings, we demonstrated that PALS-22 also inhibits the antiviral RNAi pathway, which functions to silence foreign genetic elements. Given the key roles of PALS-22 in regulating innate immunity and RNAi—two highly evolutionarily pliable systems—it is perhaps not surprising that the locus containing *pals-22* (and linked *pals-25*) is highly variable among *C. elegans* wild isolates.^96^ This variability could stem from the host-pathogen evolutionary arms race, which shapes immune responses against different natural pathogens and facilitates niche adaption.^96 ,97^

**Figure 6.**
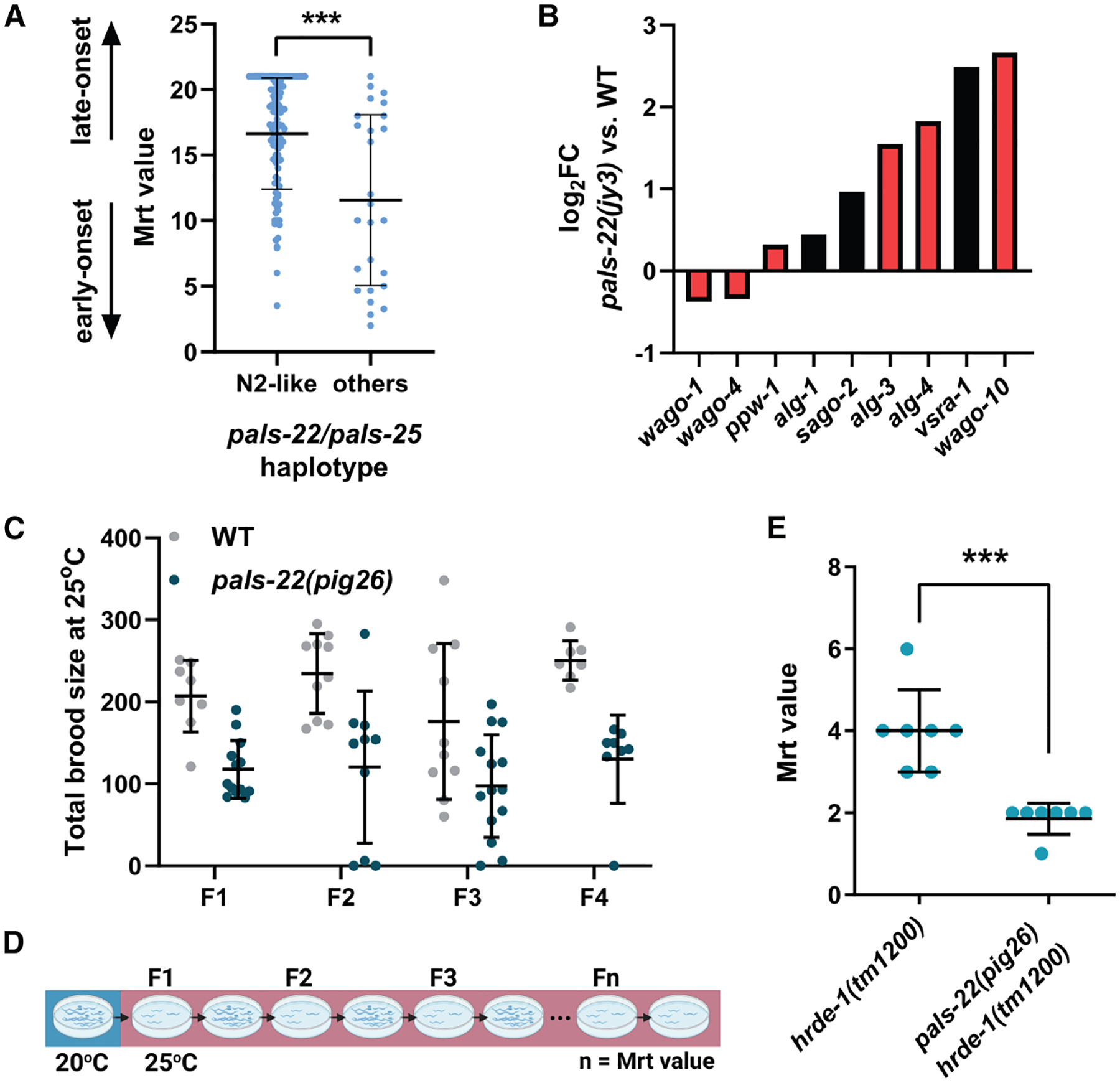
PALS-22 regulates germline mortality (A) Mrt value (mean number of generations to sterility) in wild isolates carrying either N2-like or alternate haplotypes. (B) Differential expression of *ago* genes in *pals-22 (jy3)* mutants (L4/young adults).^47^ Black and red bars indicate somatic and germline AGOs, respectively. *q* < 0.01. (C) Total brood size of wild-type and *pals-22 (pig26)* mutants at 25°C. (D) A schematic diagram illustrating the Mrt assay. Mrt value is defined as the number of generations until sterility at 25°C. (E) Mrt value of *hrde-1(tm1200)* and *pals-22(pig26) hrde-1(tm1200)* mutants. Each dot represents a biological replicate. For (A) and (E), statistical significance was determined by Mann-Whitney test. Error represents mean ± SD. ***p ≤ 0.001.

A recent study showed that many wild isolates growing at elevated temperatures (25°C) exhibit the Mrt phenotype, characterized by progressive loss of fertility, a phenomenon typically associated with dysregulation of sRNA inheritance and/or chromatin modifications. Interestingly, infection by microsporidia could rescue germline mortality in wild isolates, suggesting an interaction between the innate immunity and epigenetic inheritance processes.^27^ Therefore, we asked whether PALS-22 might influence germline mortality and transgenerational effects. We found that natural variation in *pals-22/pals-25* locus in wild isolates correlates with the penetrance of the Mrt phenotype. Strains carrying alternate haplotypes, resulting in loss of function of *pals-22* and/or *pals-25*, exhibit a more severe Mrt phenotype (a lower mean number of generations until they reach sterility at 25^◦^ C) compared with strains with the “wild-type” haplotype found in the lab reference N2 strain (Mann-Whitney test *p* = 0.0002; Figure 6A).

Similar to our findings on VSRA-1 and SAGO-2, we found that loss of *pals-22* causes dysregulation of multiple genes encoding for germline AGOs, including downregulation of WAGO-1 and WAGO-4, which are known to play roles in epigenetic inheritance (Figure 6B). Although the loss of *pals-22* alone does not cause obvious germline mortality at 25°C (Figure 6C), eliminating *pals-22* severely enhances Mrt phenotype in *hrde-1(tm1200)* mutants, which encodes a germline WAGO that is important for sRNA inheritance (Figures 6D and 6E). Our findings indicate that, although the mild misexpression of *wagos* in *pals-22(−)* mutants does not result in an overt Mrt phenotype, it significantly exacerbates germline defects in a sensitized mutant background with a compromised RNAi inheritance mechanism. These results further strengthen the connections between the immune response and epigenetic inheritance.

In summary, our results demonstrate that PALS-22 is a rapidly evolving genetic node that controls multiple immunological programs against intracellular pathogens and genomic parasites. Natural variation in this key node likely influences how animals interact with their environments and affects their ability to transmit epigenetic information across generations. Hence, our results link host-pathogen co-evolution with transgenerational epigenetic inheritance—two processes that have major impacts on the evolutionary trajectory of the animals.

## DISCUSSION

Many previous studies on *C. elegans* AGOs have primarily focused on their roles in germline development and fertility. It was found that AGO localization to perinuclear liquid-like germ granules is critical for their functions in regulating germline gene expression homeostasis.^98^ In the soma, subcellular localization of the nuclear RNAi factor NRDE-3 regulates the efficacy of RNAi.^61^ Our findings suggest that the functions of AGOs may also be highly dependent on their spatiotemporal expression and stoichiometry. For example, eliminating *vsra-1* alone does not affect transgene silencing; however, when *pals-22* is eliminated, or upon viral infection, *vsra-1* is upregulated ubiquitously. VSRA-1 is one of the few WAGOs that contain DEDH slicer active site and exhibit slicing activity *in vitro*.^6,22^ It is possible that its slicing activity is uncovered only when its concentration reaches a critical threshold. Indeed, we have shown that overexpression of *vsra-1* is sufficient to cause silencing of repetitive transgene. In mammals, for example, the loading of miRNAs depends on the expression levels of the different AGOs.^99^ In *C. elegans*, a recent study showed that the expression levels of ALG-1 and ALG-2, which function in the miRNA pathway, strongly affect miRNA processing and animals’ viability. Notably, overexpression of catalytically dead ALG-1/2 in *alg-1/2(−)* mutant backgrounds does not phenocopy animals with mutations in the catalytic tetrad but retains endogenous expression levels,^100,101^ suggesting that AGO functions are sensitive to molecular stoichiometry.

Most interestingly, our results suggest a potential cryptic gene expression-promoting role for SAGO-2, acting in the RRF-3dependent endo-siRNA pathway, when *pals-22* is eliminated. The molecular mechanism underlying this gene-activating activity of SAGO-2 is currently unclear. One possibility is that the endo-siRNA pathway may enhance the expression of IPR genes by silencing an IPR suppressor. It is possible that loss of *rrf-3* or *sago-2* perturbs a GRN that influences IPR gene expression indirectly. However, our observation of increased sRNAs targeting IPR genes in *pals-22(−)* mutants hints at a more direct mechanism. An alternative explanation might be that, in a wild-type background, low levels of SAGO-2 silence *zip-1* and IPR genes. Note that SAGO-2 lacks the DEDH catalytic tetrad in its PIWI domain and cannot directly initiate degradation of its mRNA targets^6^; thus, involvement of an additional endonuclease is likely. In the absence of *pals-22*, *sago-2* is upregulated, and the elevated level of SAGO-2 may alter its functions: instead of downregulating *zip-1* and IPR transcripts, it may act as a “sink,” binding to and protecting them from endonucleases, thereby promoting the expression of IPR genes. Although additional data are required to support the gene-activating role of siRNAs, we argue that such a mechanism could exponentially expand the complexity of RNAi regulatory outputs, particularly in the face of environmental stress or genetic perturbation, warranting many follow-up studies.

We and others have shown that PALS-22 modulates multiple immunological programs—including the transcriptional IPR and the antiviral RNAi—and controls many life history traits such as reproduction and lifespan,^51^ all of which directly impact on the animals’ competitive fitness. Some other *pals* genes have also been shown to regulate similar trade-offs between immunity and development.^47,102^ Curiously, a recent study showed that knocking out cGAS in mice causes depression of LINE1 transposons, induction of inflammation, and accelerated aging.^103^ This result reveals a surprising analogy, probably driven by convergent evolution between the highly divergent innate immune systems of *C. elegans* and vertebrates. Indeed, the gene regulatory architecture of IPR resembles that of type-I interferon response.^40^ Although the functions of many *pals* genes have not been studied, it is tempting to speculate that copy number of *pals* genes may underlie rapid diversification of the GRN governing RNAi and innate immunity and contribute to life history evolution in nematodes.

## RESOURCE AVAILABILITY

### Lead contact

Further Information and requests for resources and reagents should be directed to, and will be fulfilled by, the lead contact Oded Rechavi (odedrechavi@gmail.com).

### Materials availability

*C. elegans* strains generated in this study are available upon request.

### Data and code availability

- All NGS data are available through GEO, accession number GEO: GSE283649.
- This paper does not report original code.
- Any additional information required to reanalyze the data reported in this paper is available from the lead contact upon request.

## STAR★METHODS

### EXPERIMENTAL MODEL AND STUDY PARTICIPANT DETAILS

*C. elegans* strains were propagated at 20°C in standard Nematode Growth Media (NGM) seeded with *E. coli* OP50,^109^ unless stated otherwise. Worm strains used in this study were listed in key resources table.

### METHOD DETAILS

#### RNAi

RNAi feeding clones were obtained from the Vidal libraries.^110^ The bacteria were inoculated overnight at 37°C in LB containing 50 μg/ml carbenicillin. The next day, 1 mM of IPTG was added to the bacterial culture and 50–100 μL was seeded onto 60 mm NGM agar plates containing 1 mM IPTG and 25 μg/mL carbenicillin. After the seeded plates were dried for about 24 hours, 5–10 L4 animals were placed on the RNAi plates. The progeny were then collected for analyses.

#### Imaging and fluorescence quantification

To assess the transgene-silencing phenotype, we examined the expression of pan-neuronally expressed repetitive transcriptional reporters *otIs356 [rab-3p(prom1)::2xNLS::TagRFP]* or *otIs381 [ric-19p(prom6)::2xNLS::GFP + elt-2::DsRed]* as described previously.^51^ These repetitive transgenes were integrated by gamma radiation and the resulting strains were outcrossed extensively.^62^ The animals were immobilized with 5 mM levamisole and mounted on 2% agarose pads. Fluorescence Images were taken using Olympus IX83 motorized inverted wide-field microscope or Olympus BX63 motorized upright wide-field microscope. Transgene expression was analyzed using Fiji/ImageJ software and genotypes were blinded from the investigator using the software DoubleBlind. Corrected total worm fluorescence = integrated density – (area of selected cell X mean fluorescence of background readings). Overlapping worms and worms on edges of the image were discarded from analysis.

#### RNA sequencing

Gravid adults were treated with alkaline hypochlorite solution and the embryos were thoroughly washed and incubated in M9 overnight to obtain a synchronized L1 population. Total RNA was isolated from L1 animals using standard phenol-chloroform method using TRIzol or Qiagen RNeasy Kits per manufacture instruction.

Libraries for RNA-seq were prepared using NEBNext^®^ Ultra II Directional RNA Library Prep Kit for Illumina^®^ coupled with NEBNext^®^ Poly(A) mRNA Magnetic Isolation Module. The sample quality was accessed on Agilent 2200/4150 BioAnalyzer instrument and High Sensitivity RNA ScreenTapes. The resulting cDNA libraries were pooled and paired-end sequencing was performed on the Nextseq 550/2000 platform.

For sRNA-seq, the total RNA was first treated with RNA 5′ Polyphosphatase (epicentre) followed by library preparation using NEBNext^®^ sRNA Library Prep Set for Illumina^®^. Size selection on E-gel (Invitrogen, Life Technologies) was performed to enrich for 140–160 nt long cDNA. The sample quality was then accessed on Agilent 2200/4150 BioAnalyzer instrument and High Sensitivity RNA ScreenTapes. The sampled were pooled and sequenced on the Nextseq 550/2000 platform.

#### Bioinformatics

All bioinformatic analyses were performed using RNAlysis.^106^ For sRNA sequencing datasets, we filtered out reads < 15 and > 30 nucleotides after adapter trimming using CutAdapt.^111^ We aligned sRNA reads to PRJNA13758 ce11 genome assembly using ShortStack^112^ and counted aligned sense and anti-sense sRNA reads using FeatureCounts (Data S1).^113^ For mRNA sequencing datasets, we pseudo-aligned reads using Kallisto (Data S2).^114^ For sRNA-seq analysis (except Figures 3A and 3B), we included all genome-mapping antisense reads with > 5 RPM, without imposing constraints on sRNA length or 5′ nucleotide. We performed differential expression analysis using DESeq2.^115^ For gene set enrichment analysis, log_2_ (fold enrichment) scores were calculated and the FDR for enrichment was calculated using 10,000 random gene sets identical in size to the tested group.

#### qPCR

mRNA was isolated from L1 animals using Qiagen RNeasy Kits, except for Figures 5L, 5M, S4C, and S5F, where RNA was extracted from L4/young adults. cDNA was generated using High-Capacity cDNA Reverse Transcription Kit and qPCR was performed using 2x qPCRBIO SyGreen Blue Mix Lo-ROX. The 2–ΔΔCt method was used to calculate the relative fold-change of the samples. qCPR primers used are provided in Table S3.

#### CRISPR/Cas9 gene editing

CRISPR/Cas9 edits were performed as previously described.^116^ *pig26* deletion removes a 253 bp region spanning exons 1 and 2 of the *pals-22* gene, resulting in the loss of 69 amino acids from the protein without causing a frameshift. The mutant was backcrossed to N2 twice to eliminate potential background mutations. We have confirmed that *pig26* exhibits behaviors consistent with other *pals-22* loss-of-function mutations reported in the literature,^47,51,83^ indicating that it is a putative loss-of-function mutation.

#### Transgenesis

*pigEx48 [sago-2p::sago-2::GFP::unc-54 3’UTR + rol-6(su1006)]* extrachromosomal array was generation using a PCR fusion approach. *sago-2* gene fragment, including a 1926 bp promoter region, was fused to *gfp* and *unc-54* 3’UTR which were amplified from pPD95.75 plasmid. 25 ng/μl of the purified PCR product and 100 ng/μl of pRF4 plasmid were injected into N2. *pigEx48* shows ~37 % transmission rate.

#### Mortal germline assay

Mortal germline assay was performed as previously described.^27,117^ Briefly, four L4 animals were transferred from 20°C to 25°C (labeled F1). At every generation, four L4 animals were transferred to fresh seeded NGM plate until the population extinct. The number of generations to sterility, termed “Mrt value”, was recorded.

#### Movement tracking

20–30 L4 worms were placed on NGM plates seeded with OP50. The worms were recorded on the following three days. Each day, they were recorded for a duration of 5 mins, capturing two frames per second using Teledyne Dalsa M2420 monochrome camera. Worm motility was subsequently analyzed using the Fiji/ImageJ wrMTrck plugin (http://www.phage.dk/plugins/wrmtrck.html).

#### Brood size assay

Transfer individual L4 animal onto NGM plates seeded with OP50. The worms were transferred to fresh plates daily and the number of progeny laid each day was scored for 4–5 days, until the worms are sperm depleted.

#### *N. parisii* and Orsay virus infection assays

*N. parisii* spores were isolated as previously described.^97^ 1200 synchronized L1 animals were mixed with 5.5 × 10^5^ *N. parisii* spores and 25 μL of 10X concentrated OP50–1 bacteria, and then suspended to a total volume of 300 μL in M9 buffer. This mixture was top-plated onto unseeded 6 cm NGM plates in duplicate per condition, allowed to dry for 30 minutes at RT, and then incubated at 25°C for a 3-hour infection. Worms were washed off plates and fixed in 4% PFA prior to incubation with a FISH probe specific to *N. parisii* ribosomal RNA conjugated to a Cal Fluor 610 fluorophore. Pathogen load was determined by quantifying the number of sporoplasms present per animal across 100 worms per condition. Quantification was performed using an AxioImager under a 40X objective lens.

Infectious Orsay virus filtrate (OVF) was prepared as previously described.^118^ 2000 synchronized L1 animals were plated onto 10 cm NGM plates seeded with 500 μL of 10X OP50–1 and allowed to grow to L4 stage (44 hours at 20°C), after which an infection mix comprised of 150 μL of 10x OP50–1, 30 μL of OVF, and 720 μL of M9 (900 μL total per plate) was top-plated. Once dry, plates were incubated at 20°C for 24 hours prior to fixation in 4% PFA and incubation with two FISH probes specific to both Orsay virus RNA1 and RNA2, each conjugated to a Cal Fluor 610 fluorophore. Images of the FISH probe signal across approximately 15 worms per condition were taken using an AxioImager under a 10X objective, and the signal was then quantified using ImageJ, correcting for background fluorescence.

### QUANTIFICATION AND STATISTICAL ANALYSIS

Statistical analyses were conducted using R software v4.2.3 and GraphPad Prism 10. Parametric tests were used for data with a normal distribution and equal variances, while non-parametric tests were applied when the assumptions were not met. Multiple comparison corrections using the Benjamini–Hochberg method were applied where appropriate. Details of the statistical tests and the number of replicates are provided in the figure legends.

## Supplementary Material

Data S1

Data S2

1

Supplemental information can be found online at https://doi.org/10.1016/j.cub.2025.05.039.

## Figures and Tables

**Table:** KEY RESOURCES TABLE

REAGENT or RESOURCE	SOURCE	IDENTIFIER
Bacterial and virus strains		
OP50	Caenorhabditis Genetics Center	N/A
OP50–1	Caenorhabditis Genetics Center	N/A
HT115	Caenorhabditis Genetics Center	N/A
Orsay virus	Félix et al.^50^	N/A
Chemicals, peptides, and recombinant proteins		
Alt-R^™^ S.p. Cas9 Nuclease V3	IDT	Cat#1081058
Critical commercial assays		
NEBNext^®^ Small RNA Library Prep Set for Illumina^®^	New England Biolabs	Cat#E7330L
NEBNext^®^ Ultra^™^ II Directional RNA Library Prep Kit for Illumina^®^	New England Biolabs	Cat#E7760, #E7765
High-Capacity cDNA Reverse Transcription Kit	Applied Biosystems	Cat#4368814
2x qPCRBIO SyGreen Blue Mix Lo-ROX	PCR Biosystems	Cat#PB20.15–01
RNeasy Mini Kit	Qiagen	Cat#74104
Deposited data		
Raw and analyzed sequencing data	This paper	GEO: GSE283649
adr-1/2(−) sequencing data	Fischer and Ruvkun^104^	GEO: GSE143595
AGO IP sequencing data	Seroussi et al.^1^	GEO: GSE208702
Experimental models: Organisms/strains		
*C. elegans* strain: BFF144 pals-22(pig26) III	This study	BFF144
*C. elegans* strain: BFF151 pals-22(pig26) III; otIs356 [rab-3p(prom1)::2xNLS::TagRFP] V line 1	This study	BFF151
*C. elegans* strain: BFF152 pals-22(pig26) III; otIs356 [rab-3p(prom1)::2xNLS::TagRFP] V line 2	This study	BFF152
*C. elegans* strain: BFF156 set-25(n5021) III; otIs356 [rab-3p(prom1)::2xNLS::TagRFP] V	This study	BFF156
*C. elegans* strain: BFF178 pals-22(pig26) II; set-25(n5021) III; otIs356 [rab-3p(prom1)::2xNLS::TagRFP] V	This study	BFF178
*C. elegans* strain: BFF189 otIs356 [rab-3p(prom1)::2xNLS::TagRFP] V; nrde-3(tm1116) X	This study	BFF189
*C. elegans* strain: BFF191 pals-22(pig26) II; otIs356 [rab-3p(prom1)::2xNLS::TagRFP] V; nrde-3(tm1116) X	This study	BFF191
*C. elegans* strain: BFF192 rrf-1(ok589) I; otIs356 [rab-3p(prom1)::2xNLS::TagRFP] V	This study	BFF192
*C. elegans* strain: BFF208 drh-1(ok3495) IV; otIs356 [rab-3p(prom1)::2xNLS::TagRFP] V	This study	BFF208
*C. elegans* strain: BFF217 pals-22(pig26) II; drh-1(ok3495) IV; otIs356 [rab-3p(prom1):: 2xNLS::TagRFP] V line 1	This study	BFF217
*C. elegans* strain: BFF218 pals-22(pig26) II; drh-1(ok3495) IV; otIs356 [rab-3p(prom1)::2xNLS::TagRFP] V line 2	This study	BFF218
*C. elegans* strain: BFF225 rrf-1(ok589) I; pals-22(pig26) II; otIs356 [rab-3p(prom1)::2xNLS::TagRFP] V line 1	This study	BFF225
*C. elegans* strain: BFF226 rrf-1(ok589) I; pals-22(pig26) II; otIs356 [rab-3p(prom1)::2xNLS::TagRFP] V line 2	This study	BFF226
*C. elegans* strain: BFF294 rrf-3(pk1426) II; pals-22(pig26) III; otIs356 [rab-3p(prom1)::2xNLS::TagRFP] V	This study	BFF294
*C. elegans* strain: BFF295 pals-22(pig26) III; nrde-3(tor131[GFP::3xFLAG::nrde-3]) X.	This study	BFF295
*C. elegans* strain: BFF296 rrf-3(pk1426) II; otIs356 [rab-3p(prom1)::2xNLS::TagRFP] V	This study	BFF296
*C. elegans* strain: BFF297 rrf-3(pk1426) II; pals-22(pig26) III	This study	BFF297
*C. elegans* strain: N2 WT control	Caenorhabditis Genetics Center	WB Strain: N2
*C. elegans* strain: NL2099 rrf-3(pk1426) II	Caenorhabditis Genetics Center	WB Strain: NL2099
*C. elegans* strain: OH10690 otIs356 [rab-3p(prom1)::2xNLS::TagRFP] V	Caenorhabditis Genetics Center	WB Strain: 0H10690
*C. elegans* strain: BFF180 pals-22(pig26) III; gg547[pgl-1::3xFLAG::TagRFP] IV	This study	BFF180
*C. elegans* strain: YY967 pgl-1(gg547[pgl-1::3xFLAG::TagRFP]) IV.	Wan et al.^105^	YY967
*C. elegans* strain: BFF375 vsra-1(tor94[GFP::3xFLAG::vsra-1] I; pals-22(pig26) III; otIs356 [rab-3p(prom1)::2xNLS::TagRFP] V	This study	BFF375
*C. elegans* strain: BFF374 vsra-1(tor94[GFP::3xFLAG::vsra-1] I; pals-22(pig26) III	This study	BFF374
*C. elegans* strain: BFF368 pals-22(pig26) III; otIs356 [rab-3p(prom1)::2xNLS::TagRFP] V; sid-4(ok694) X	This study	BFF368
*C. elegans* strain: BFF367 sago-2(tm894) I; pals-22(pig26) III; otIs356 [rab-3p(prom1)::2xNLS::TagRFP] V	This study	BFF367
*C. elegans* strain: BFF366 adr-2(gv42) pals-22(pig26) III; otIs356 [rab-3p(prom1)::2xNLS::TagRFP] V	This study	BFF366
*C. elegans* strain: BFF365 adr-2(gv42) III; otIs356 [rab-3p(prom1)::2xNLS::TagRFP] V	This study	BFF365
*C. elegans* strain: BFF363 otIs356 [rab-3p(prom1)::2xNLS::TagRFP] V; sid-4(ok694) X	This study	BFF363
*C. elegans* strain: BFF349 pals-22(pig26) III; otIs356 [rab-3p(prom1)::2xNLS::TagRFP] V; sid-3(ok973) X	This study	BFF349
*C. elegans* strain: BFF345 otIs356 [rab-3p(prom1)::2xNLS::TagRFP] V; sid-3(ok973) X	This study	BFF345
*C. elegans* strain: BFF344 vsra-1(tm1637) I; pals-22(pig26) III; otIs356 [rab-3p(prom1):: 2xNLS::TagRFP] V	This study	BFF344
*C. elegans* strain: BFF343 vsra-1(tm1637) I; otIs356 [rab-3p(prom1)::2xNLS::TagRFP] V	This study	BFF343
*C. elegans* strain: JMC135 vsra-1(tor94[GFP::3xFLAG::vsra-1] I.	Caenorhabditis Genetics Center	WB Strain: JMC135
*C. elegans* strain: WM154 sago-2(tm894)	Caenorhabditis Genetics Center	WB Strain: WM154
*C. elegans* strain: JMC227 sago-2(tor121[GFP::3xFLAG::sago-2c]) I.	Caenorhabditis Genetics Center	WB Strain: JMC227
*C. elegans* strain: BFF439 sago-2(tor121[GFP::3xFLAG::sago-2c]) I.; zip-1(jy14) III	This study	BFF439
*C. elegans* strain: BFF169 hrde-1(tm1200) III; otIs356 [rab-3p(prom1)::2xNLS::TagRFP] V.	This study	BFF169
*C. elegans* strain: BFF170 pals-22(pig26) hrde-1(tm1200) III; otIs356 [rab-3p(prom1)::2xNLS::TagRFP] V.	This study	BFF170
*C. elegans* strain: BFF411 vsra-1(tor94[GFP::3xFLAG::vsra-1] I; zip-1(jy14) III	This study	BFF411
*C. elegans* strain: BFF405 vsra-1(tor94[GFP::3xFLAG::vsra-1] I; pals-22/25(jy79) III	This study	BFF405
*C. elegans* strain: OH11061 otIs381 [ric-19p(prom6)::2xNLS::GFP + elt-2::DsRed] V	Caenorhabditis Genetics Center	WB Strain: OH11061
*C. elegans* strain: BFF415 vsra-1(tm1637) I; pals-22(pig26)III; otIs381 [ric-19p(prom6)::2xNLS::GFP + elt-2::DsRed] V	This study	BFF415
*C. elegans* strain: BFF418 pals-22(pig26) III; otIs381 [ric-19p(prom6)::2xNLS::GFP + elt-2::DsRed]	This study	BFF418
*C. elegans* strain: BFF459 fjSi19 [rpl-21p::2×HA::vsra-1 + Cbr-unc-119(+)] II; otIs356 [rab-3p(prom1)::2xNLS::TagRFP] IV	This study	BFF459
*C. elegans* strain: WM158 ergo-1(tm1860) V	Caenorhabditis Genetics Center	WB Strain: WM158
*C. elegans* strain: BFF480 pigEx48[sago2p::SAGO-2::GFP::unc-543’UTR +rol-6(su1006)]	This study	BFF480
*C. elegans* strain: BFF490 neIs10 [myo-3::GFP::sago-2 + rol-6(su1006)]	This study	BFF490
*N. parisii* strain: ERTm1	Troemel et al.^49^	ERTm1
Oligonucleotides		
pals-22(pig26) Alt-R gRNA #1: TGGCCTGCAAGACGAGGTAC	IDT	N/A
pals-22(pig26) Alt-R gRNA #2: GGTTTGGGAAGAACTTAAAA	IDT	N/A
pals-22(pig26) Alt-R HDR donor block: CAGGTTGCATAGAGAAGAAGATGAACTCGCCGGTAAAAAGGTGTTGGAAGCGGCTGAAATAGTTGATGTG	IDT	N/A
Custom FISH probes for *N. parisii* ribosomal RNA	Biosearch Technologies	N/A
Custom FISH probes for Orsay virus RNA1 and RNA2	Biosearch Technologies	N/A
See Table S3 for genotyping primer sequences used in this study	IDT	N/A
Recombinant DNA		
Plasmid: pL4440-RNAi control (HT115)	Vidal RNAi library	N/A
Plasmid: pL4440-pals-22 (HT115)	Vidal RNAi library	N/A
Plasmid: pL4440-zip-1 (HT115)	Vidal RNAi library	N/A
Plasmid: pPD95.75	Gift from Zaidel-Bar lab	Addgene Plasmid #1494
Software and algorithms		
RNAlysis	Teichman et al.^106^	https://github.com/GuyTeichman/RNAlysis
ImageJ	Schindelin et al.^107^	https://imagej.net/ij/
GraphPad Prism 10	GraphPad	https://www.graphpad.com/
R/RStudio	R Core Team	https://www.r-project.org/
BioRender	BioRender	https://www.biorender.com/
DoubleBlind	Teichman et al.^108^	https://github.com/GuyTeichman/DoubleBlind
Inkscape v1.4	Inkscape	https://inkscape.org/

## Highlights

- PALS-22, a rapidly evolving genetic factor, negatively regulates antiviral RNAi
- PALS-22 inhibits the expression of AGOs by suppressing ZIP-1/bZIP TF
- PALS-22 modulates immune gene expression by regulating the endo-siRNA pathway
- PALS-22 affects epigenetic inheritance and contributes to germline immortality
