# Supplementary material for: Dynamic control of Argonautes by a rapidly evolving immunological switch
Source: Curr Biol. Author manuscript; Available in PMC 2025 Sep 1. (PMC12401492; doi:10.1016/j.cub.2025.05.039)
Supplement: 1 [file NIHMS2105966-supplement-1.pdf]

Current Biology, Volume *35*

Supplemental Information

Dynamic control of Argonautes by a rapidly evolving immunological switch

Chee Kiang Ewe, Guy Teichman, Shir Weiss, Maximilian M.L. Knott, Sarit Anava, Hila Gingold, Mario Bardan Sarmiento, Emily Troemel, and Oded Rechavi

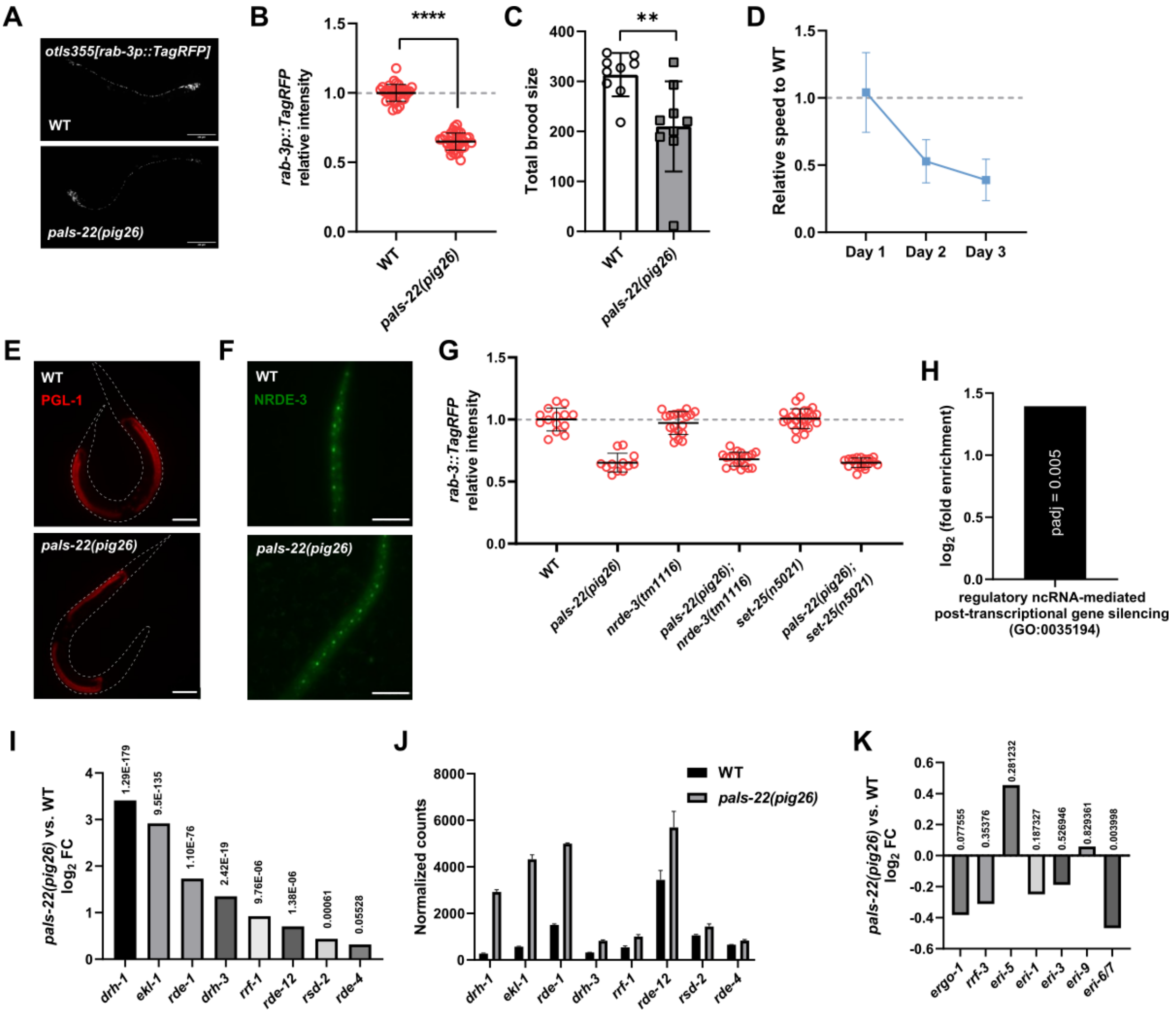

Figure S1: Loss of *pals-22* results in enhanced antiviral RNAi, related to Figure 1.

(A, B) Expression of *otIs356 [rab-3p::TagRFP]* in wild type and *pals-22(pig26)* mutants. Scale bar = 100 µm (C) Brood size of wild type and *pals-22(pig26)* mutants at 20°C. (D) Locomotion speed of *pals-22(pig26)* mutants normalized to aged matched wild-type animals. Error represents mean +/- SD. Statistical significance was determined by two-tailed unpaired t-tests. ** ≤ 0.01; **** p ≤ 0.0001. (E) The expression of *pgl-1::TagRFP* in wild-type and *pals-22(pig26)* day 1 adults. Scale bar = 100 µm. (F) The expression of endogenous NRDE-3::GFP reporter in wild-type and *pals-22(pig26)* L2 animals. Scale bar = 10 µm. (G) The expression repetitive *rab-3::TagRFP* transgene in *pals-22(pig26)* and nuclear RNAi mutants. Each data point represents an animal scored. Error represents mean +/- SD. (H) GO term enrichment of genes upregulated in *pals-22(pig26)* vs. wild type. (I-K) RNAi genes expression in *pals-22(pig26)* vs. wild type. Numbers in panels (I) and (K) indicate DESeq2 q-values.

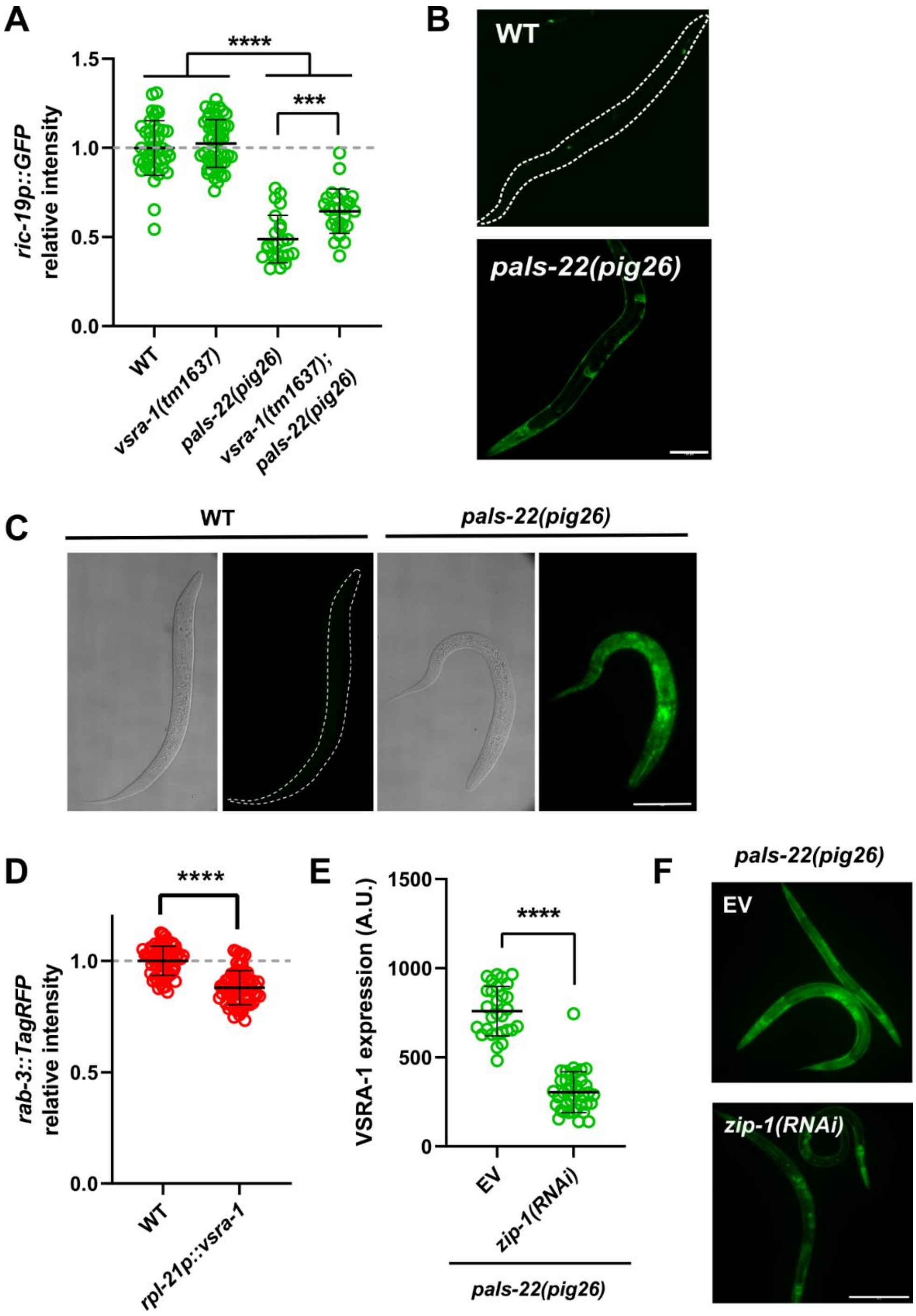

Figure S2: PALS-22 represses VSRA-1, related to Figure 2.

(A) The interaction between *pals-22* and *vsra-1* in silencing *otIs381 [ric-19p(prom6)::2xNLS::GFP + elt-2::DsRed]* transgene. Statistical significance was determined by parametric one-way ANOVA followed by pairwise two-tailed unpaired t-tests with Benjamini–Hochberg correction. *** q ≤ 0.001; **** q ≤ 0.0001. Error represents mean +/- SD. Each data point represents an animal scored. (B) The expression of endogenous VSRA-1 in wild-type and *pals-22(pig26)* day 1 adults. Scale bar = 100 μm. (C) The

expression of endogenous VSRA-1 in wild-type and *pals-22(pig26)* L1 animals. Scale bar = 50 µm. (D) The expression repetitive *rab-3::TagRFP* transgene in wild-type and mutants carrying *fjSi19 [rpl-21p::2×HA::C04F12.1 + Cbr-unc-119(+)]*. Each data point represents an animal scored. Statistical significance was determined by two-tailed unpaired t-test. **** $p \leq 0.0001$. Error represents mean +/- SD. (E, F) The expression of VSRA-1 in *pals-22(pig26)* animals treated with empty vector or *zip-1* RNAi. Each data point represents an animal scored. Statistical significance was determined by two-tailed unpaired t-test. **** $p \leq 0.0001$. Error represents mean +/- SD. Scale bar = 200 µm.

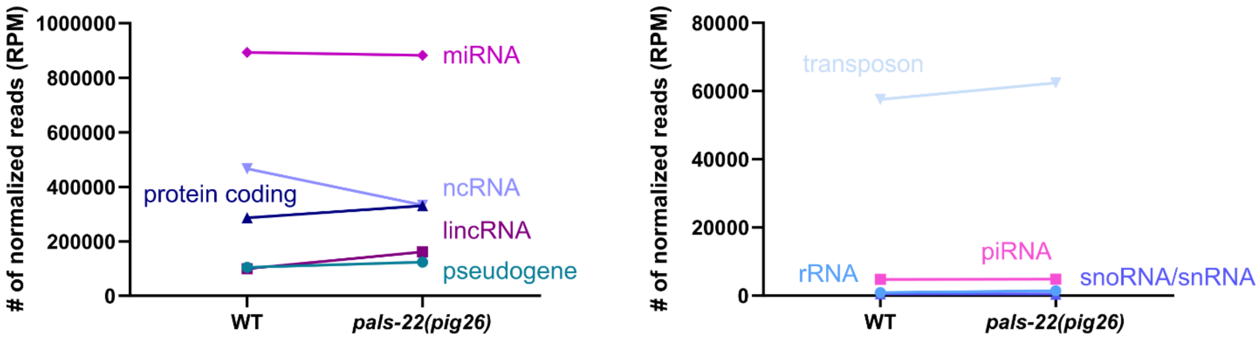

Figure S3: Biotypes of small RNAs isolated from wild-type and *pals-22(pig26)* animals, related to Figure 3.

Sense (miRNA and piRNA) and antisense (others) small RNAs aligned to different gene biotypes. All mapped reads > 5 RPM were counted without any constraints on small RNA length or 5′ nucleotide. RPM: read per million

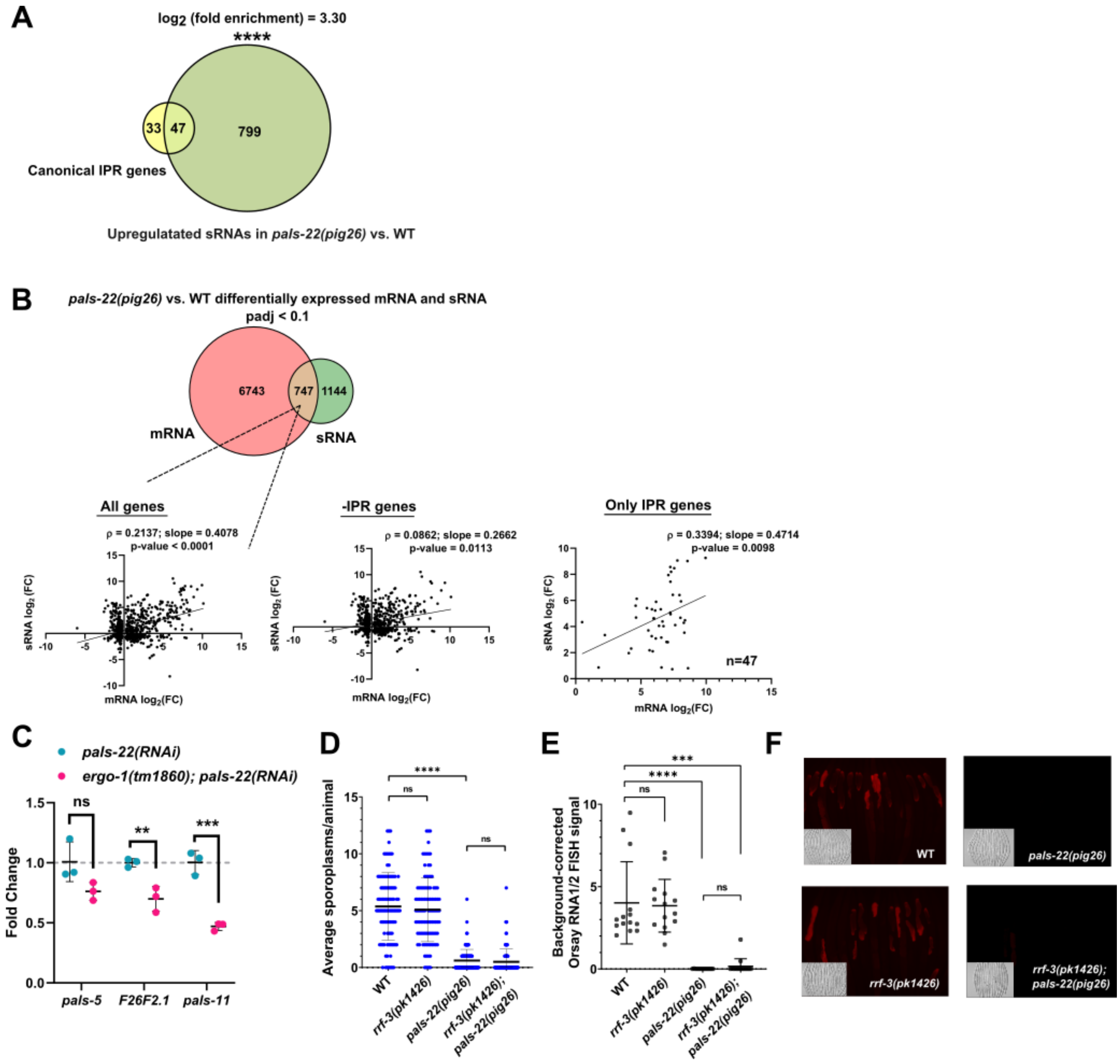

Figure S4: Dual effects of the endo-siRNA pathway on IPR gene expression, related to Figure 4.

(A) Venn diagram showing the overlap between 80 canonical IPR genes and small RNA upregulated in *pals-22(pig26)* vs. wild type. (B) Spearman correlation between differentially expressed mRNA and small RNA in *pals-22(pig26)* vs. wild type. The lines are fitted with linear regression model. (C) The expression of IPR genes in *pals-22(RNAi)* and *ergo-1(tm1860); pals-22(RNAi)* young adults, as measured by qPCR. Statistical significance was determined by two-tailed unpaired t-test. ns, not significant ($p > 0.05$); ** $p \leq 0.01$; *** $p \leq 0.001$. Error represents mean +/- SD. (D) Sensitivity of *rrf-3(pk1426)*, *pals-22(pig26)*, and *rrf-3(pk1426); pals-22(pig26)* mutants to *N. parisii* infection. (E) Sensitivity of *rrf-3(pk1426)*, *pals-22(pig26)*, and *rrf-3(pk1426); pals-22(pig26)* mutants to Orsay virus infection. Statistical significance was determined by non-parametric Kruskal–Wallis test followed by

pairwise Wilcoxon Rank Sum tests with Benjamini Hochberg correction. ns, not significant ($q > 0.05$); *** $q \leq 0.001$; **** $q \leq 0.0001$. Error represents mean +/- SD. (F) Representative micrographs of animals from (E).

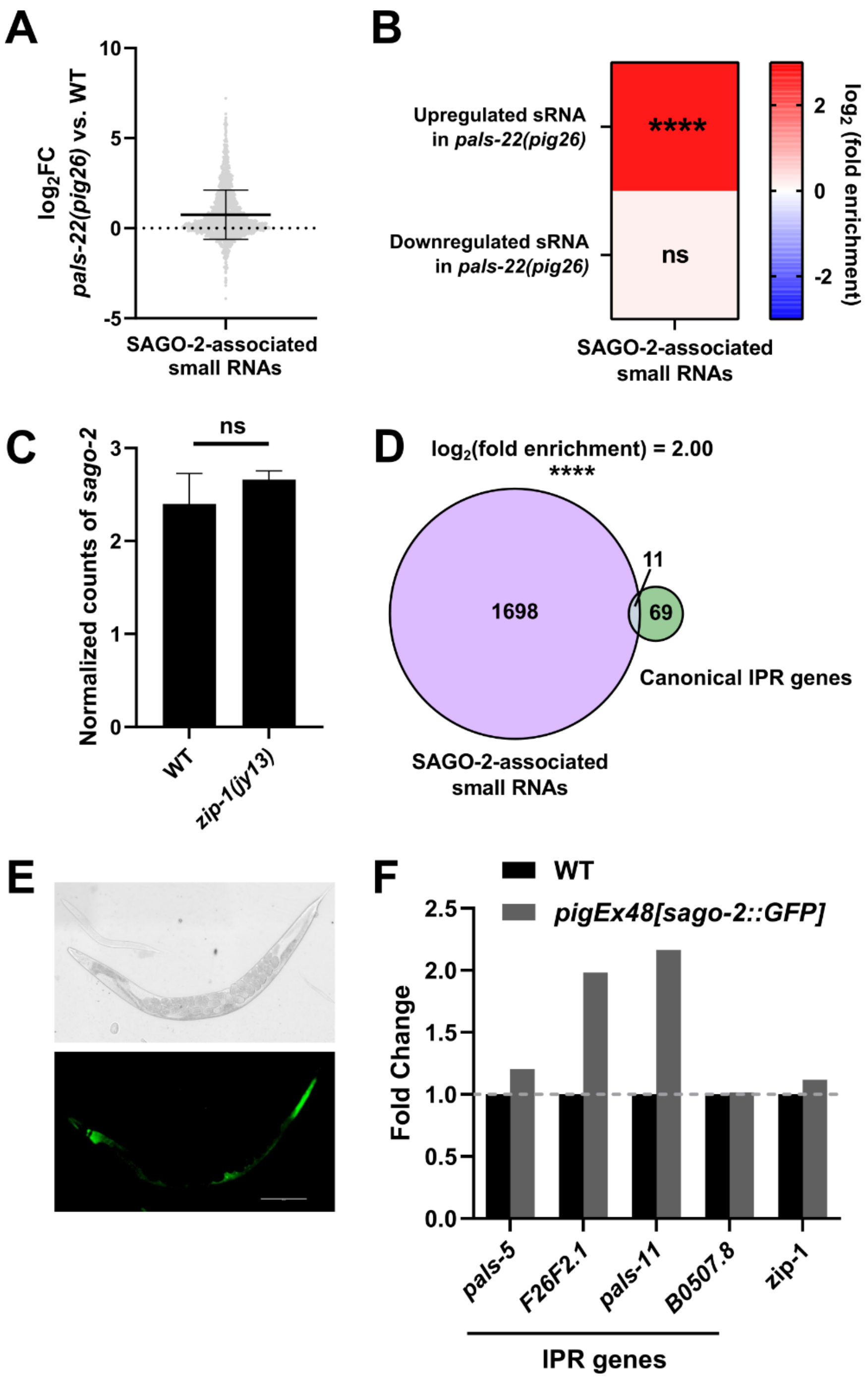

Figure S5: PALS-22 regulates SAGO-2, which targets IPR genes, related to Figure 5.

(A) The expression of SAGO-2-associated small RNAs in *pals-22(pig26)* vs. wild type. (B) Enrichment analysis of SAGO-2-associated small RNAs in small RNAs differentially expressed in *pals-22(pig26)* vs. wild type. ns, not significant (q > 0.05); **** q ≤ 0.0001. (C) Expression of *sago-2* in wild type and *zip-1(jy13)* animals detected by RNA-seq. ns, not significant (q > 0.05). The data was extracted from

Lažetić, *et. al.* S1 (D) Venn diagram showing the overlap between 80 canonical IPR genes and SAGO-2-associated small RNAs. (E) The expression of *pigEx48 [sago-2::GFP::unc-54 3'UTR+rol-6(su1006)]* in day 1 adult. Scale bar = 200 μm. (F) The expression of IPR genes and *zip-1* in wild type and animals carrying *pigEx48[sago-2::GFP::unc-54 3'UTR+rol-6(su1006)].*

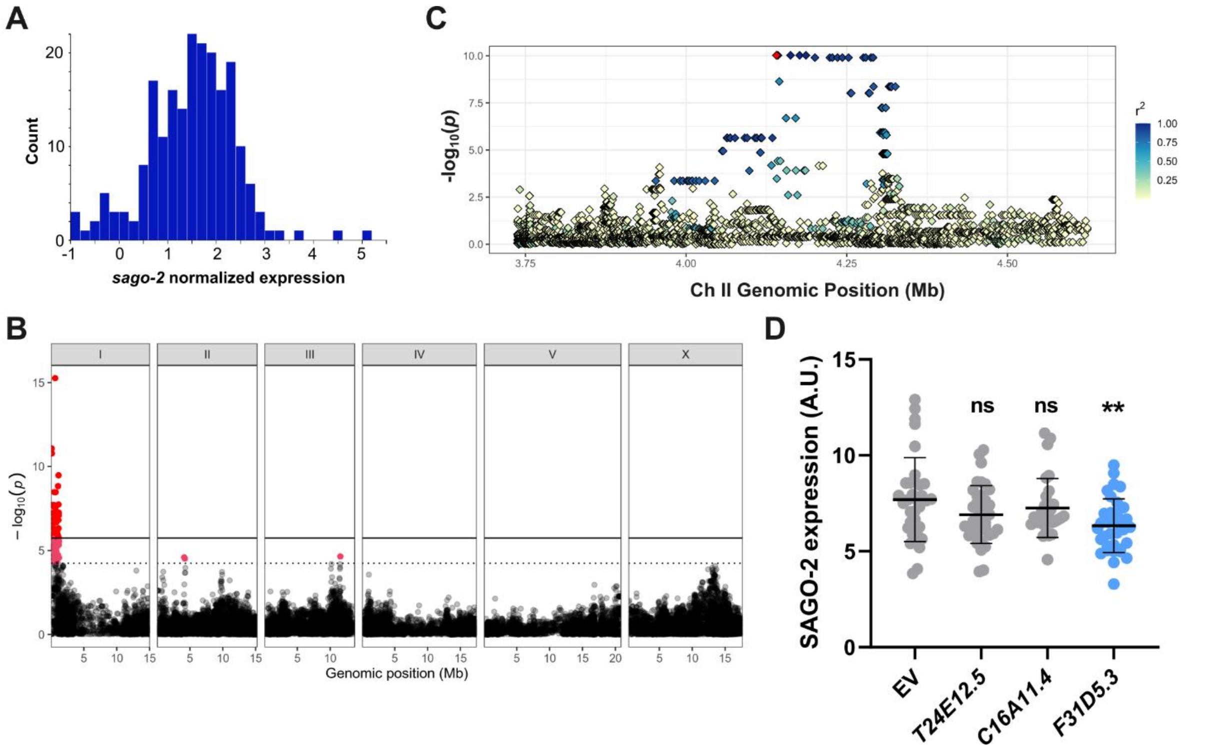

Figure S6: Natural variation in *cpna-1/F31D5.3* may affect *sago-2* expression, related to Figure 5.

(A) Natural variation of *sago-2* expression in 207 *C. elegans* wild isolates. (B) GWAS using LOCO kinship matrix. The horizontal solid line corresponds to stricter Bonferroni (BF) threshold, while the horizontal dash line corresponds to more permissive EIGEN threshold. Red dots represent statistically significant SNPs. (C) Fine mapping of Chr 2 candidate region (II:3737765-4624311). Each variant is represented by a diamond colored by the linkage to the peak marker (colored in red). GWAS was performed on CaeNDR (https://caendr.org/). (D) Targeted RNAi screen focusing on candidate genes in Chr 2. Each data point represents an animal scored. Statistical significance was determined by non-parametric Kruskal–Wallis test followed by pairwise Wilcoxon Rank Sum tests with Benjamini–Hochberg correction. ns, not significant ($q > 0.05$); ** $q \leq 0.01$, compared to EV.

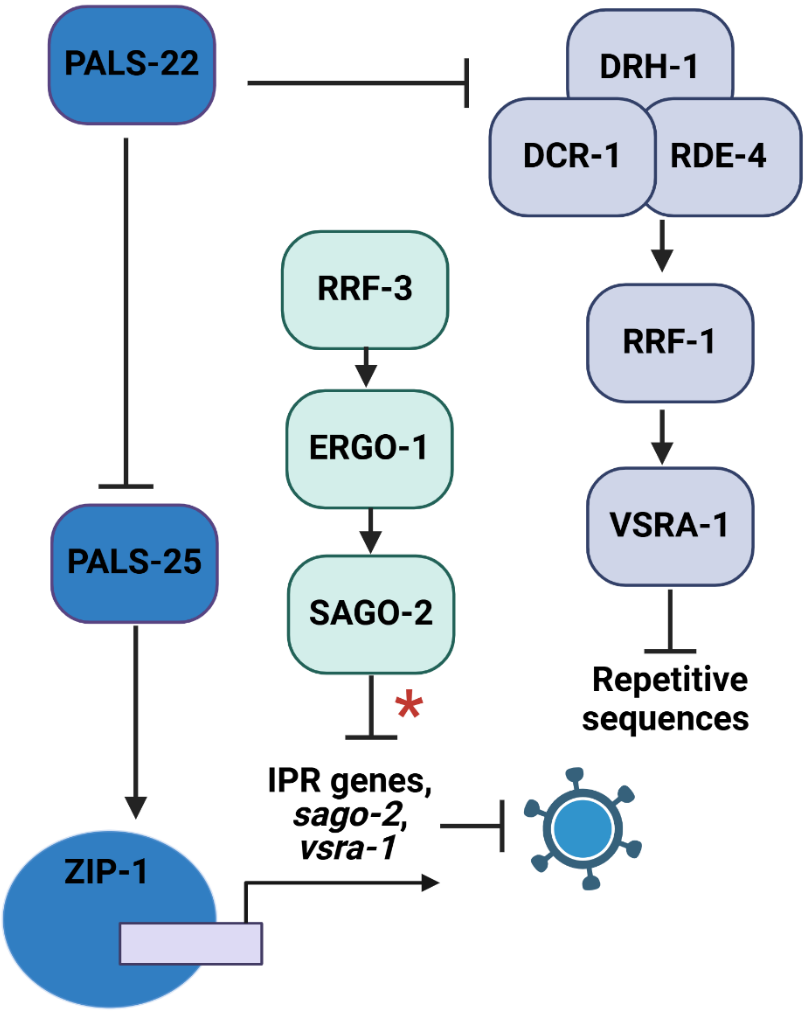

Figure S7: A model of PALS-22 regulation of the small RNA pathway, related to Figure 5.

PALS-22 suppresses the IPR and antiviral RNAi pathway. The endo-siRNA pathway, acting through SAGO-2, may positively or negatively regulate IPR genes and *zip-1* expression, depending on PALS-22 activity.

Tissue	Scaled TPM
Unassigned sheath cells	90.95
AVL	57.83
Glia_3	54.23
sh3_sh4 (gonadal sheath proximal)	52.94
Oocytes	44.99
Amphid sheath	37.31
AWB	36.56
Spermathecal-Uterine junction	36.11
Coelomocytes	30.49
RIR	23.35
Phasmid sheath	22.33
Dorsal uterine cell	19.45
AVM	18.95
RMD_LR	8.90
Intestinal-rectal valve	4.36
g2	2.30
Spermathecal bag distal	1.12

Table S1: Expression of *vsra-1* based on single-cell sequencing of young adult worm (WormSeq [S2]). Only tissues with detectable expression are shown below. TPM = transcript per million. Related to Figure 2.

Category	Number of genes	All catalog values	p-value	Bonferroni
**Downregulated siRNAs in *pals-22(pig26)* vs. WT**				
Proteolysis proteasome: E3: F box	55	437	6.08E-15	1.17E-12
Pseudogene	124	2081	1.61E-08	3.09E-06
Unassigned	211	4113	5.15E-08	9.88E-06
**Upregulated siRNAs in *pals-22(pig26)* vs. WT**				
Unassigned: ALS2cr12 domain*	13	31	7.44E-11	1.54E-08
Proteolysis proteasome: E3: F box	37	437	1.04E-08	2.16E-06
Ribosome: subunit	16	92	2.92E-08	6.05E-06
Unassigned: regulated by multiple stresses	84	1707	1.13E-06	0.00023382
Proteolysis general: aspartate: unassigned	5	11	4.33E-05	0.00897293

**pals genes*

Table S2: GO term enrichment in siRNAs differentially expressed in *pals-22(pig26)* vs. wild type. This analysis was performed using the WormCat software.[S3] Related to Figure 3.

Name	Sequence	Description
EE41	TGGTTTACTCTAGAGGGGGAAGA	*set-25(n5021)* forward primer
EE42	CCGTGCTACGCGGTAAGTAT	*set-25(n5021)* reverse primer
EE17	GCGACTCGACCTATCAGTGC	*pals-22(pig26)* forward primer
EE18	ATTTTGCCGCCCATCCCTAA	*pals-22(pig26)* reverse primer
EE59	ACGTTTATTCCGCGTTGCAC	*nrde-3(tm1116)* forward primer
EE60	AAAAACCCACATCCTCCCAG	*nrde-3(tm1116)* reverse primer
EE75	CCGGTTAGCTGAAATTCATACTTGT	*rrf-1(ok589)* forward primer external
EE76	TGTCCCCCACTCATCAGGAA	*rrf-1(ok589)* reverse primer external
EE91	ACTCAGATTATTGAGGCAATTCTTT	*rrf-1(ok589)* forward primer internal
EE92	TTGAAGAAATCGCCGACCGA	*rrf-1(ok589)* reverse primer internal
EE87	TCCAACAGGATCCCTCCACTA	*drh-1(ok3495)* forward primer external
EE88	TTCTCCATTCGCGTGATGCT	*drh-1(ok3495)* reverse primer external
EE97	TGGCCTCGAATCCTGAGAGA	*drh-1(ok3495)* forward primer internal
EE98	CTCATCCGTCAGGCGAGTTT	*drh-1(ok3495)* reverse primer internal
EE129	ACACACTTGAAATTGGTAAGCGA	*rrf-3 (pk1426)* forward primer external
EE130	TACACTCCCGCGTGTTTCAA	*rrf-3 (pk1426)* reverse primer external
EE131	AGTTTTCGACGCAGTTTGGC	*rrf-3 (pk1426)* forward primer internal
EE132	TTCAAGGAACATACTGGAAAAGTC	*rrf-3 (pk1426)* reverse primer internal
EE135	GCTATTACATGAGTCACACC	*sago-2(tm894)* forward primer
EE136	GTCGGTTACCGGCAAACGCA	*sago-2(tm894)* reverse primer
EE137	AGCTTGGCTCGTCACAAATCT	*vsra-1(tm1637)* forward primer
EE138	GCACACTTGGTCATGGACTGT	*vsra-1(tm1637)* reverse primer
EE139	AAGTTTCCTTTCTTCTCAATGTTGT	*sid-3(ok973)* forward primer external
EE140	TTGATGGCACAGAAGACGCT	*sid-3(ok973)* reverse primer external
EE149	CAGTTTCTCAGCTCCAGGCA	*adr-2(gv42)* forward primer
EE150	ACCACATTTTTCGAGATGCTCA	*adr-2(gv42)* reverse primer #1
EE151	CTCGGTGGTGCTTCTGAGTT	*adr-2(gv42)* reverse primer #2
EE156	AAATGAAGACCGTGACGCCT	*sid-4(ok694)* forward primer
EE157	CTTCCAGCCGAAAGCAACAG	*sid-4(ok694)* reverse primer #1
EE158	ACTTGCCGAACTTAATACTTGTGT	*sid-4(ok694)* reverse primer #2
EE47	GCATCGCTTCGTGTTTCCTC	*hrde-1(tm1200)* forward primer
EE48	CCGCTCAAAATCAGTACTTTCCA	*hrde-1(tm1200)* reverse primer
EE207	GCGAAAATAGGCGTGGTATTG	*zip-1(jy14)* forward primer #1
EE208	CTTCTGGCCTTCCTCATTGAT	*zip-1(jy14)* forward primer #2
EE209	GGAGTTCAAAGTCGCTGATTG	*zip-1(jy14)* reverse primer
EE187	TAATCGTTTGTCGCCCACCT	*pals-22/25(jy79)* forward primer
EE188	GTACTGATCCGTTGCAGGCT	*pals-22/25(jy79)* reverse primer
EE212	ACGCGTTGAAAGAGAAGAATGT	*pals-5* qPCR forward primer
EE213	TTCGTTCAAAACATCGGCGT	*pals-5* qPCR reverse primer
EE214	CAGAGTGGCACAAGAACGGA	*F26F2.1* qPCR forward primer
EE215	CACCCCTCTGGGATTCATGG	*F26F2.1* qPCR reverse primer
EE216	TGCCTTAGCGGATAAGAAAAAGT	*pals-11* qPCR forward primer
EE217	CCTTTGCTTGCATTCTCATGCT	*pals-11* qPCR reverse primer
EE218	TGCCTTCGTGCCATTAACGA	*B0507.8* qPCR forward primer
EE219	TCCGGAGCTCATCACTCATTG	*B0507.8* qPCR reverse primer
EE220	TCGACAATTACGCCGTCACA	*cdc-42* qPCR forward primer
EE221	GAAACACGTCGGTCTGTGGA	*cdc-42* qPCR reverse primer
EE277	GCAGTTGGAAGAGGCCTTCA	*zip-1* qPCR forward primer
EE278	ATGGTCGGCGATCTCGAAAG	*zip-1* qPCR reverse primer
EE248	GGAGCCGATTTGTTCCAGTC	*fjSi19* forward primer
EE249	ATCGGGAGGCGAACCTAACTG	*fjSi19* reverse primer

Table S3: Genotyping primers used in this study. Related to STAR Methods.

Supplemental references

S1. Lažetić, V., Wu, F., Cohen, L.B., Reddy, K.C., Chang, Y.-T., Gang, S.S., Bhabha, G., and Troemel, E.R. (2022). The transcription factor ZIP-1 promotes resistance to intracellular infection in Caenorhabditis elegans. Nat Commun *13*, 17. https://doi.org/10.1038/s41467-021-27621-w.

S2. Ghaddar, A., Armingol, E., Huynh, C., Gevirtzman, L., Lewis, N.E., Waterston, R., and O'Rourke, E.J. (2023). Whole-body gene expression atlas of an adult metazoan. Science Advances *9*, eadg0506. https://doi.org/10.1126/sciadv.adg0506.

S3. Holdorf, A.D., Higgins, D.P., Hart, A.C., Boag, P.R., Pazour, G.J., Walhout, A.J.M., and Walker, A.K. (2020). WormCat: An Online Tool for Annotation and Visualization of Caenorhabditis elegans Genome-Scale Data. Genetics *214*, 279–294. https://doi.org/10.1534/genetics.119.302919.
